# Comparison of Coral Reef Ecosystems along a Fishing Pressure Gradient

**DOI:** 10.1371/journal.pone.0063797

**Published:** 2013-05-30

**Authors:** Mariska Weijerman, Elizabeth A. Fulton, Frank A. Parrish

**Affiliations:** 1 Joint Institute for Marine and Atmospheric Research, University of Hawai’i at Manoa, Honolulu, Hawaii, United States of America; 2 Wageningen Institute for Environment and Climate Research, University of Wageningen, Wageningen, The Netherlands; 3 Division of Marine Research, Commonwealth Scientific and Industrial Research Organisation Hobart, Tasmania, Australia; 4 Protected Species Division, Pacific Islands Fisheries Science Center, Honolulu, Hawaii, United States of America; Universidade Federal do Rio de Janeiro, Brazil

## Abstract

Three trophic mass-balance models representing coral reef ecosystems along a fishery gradient were compared to evaluate ecosystem effects of fishing. The majority of the biomass estimates came directly from a large-scale visual survey program; therefore, data were collected in the same way for all three models, enhancing comparability. Model outputs–such as net system production, size structure of the community, total throughput, production, consumption, production-to-respiration ratio, and Finn’s cycling index and mean path length–indicate that the systems around the unpopulated French Frigate Shoals and along the relatively lightly populated Kona Coast of Hawai’i Island are mature, stable systems with a high efficiency in recycling of biomass. In contrast, model results show that the reef system around the most populated island in the State of Hawai’i, O’ahu, is in a transitional state with reduced ecosystem resilience and appears to be shifting to an algal-dominated system. Evaluation of the candidate indicators for fishing pressure showed that indicators at the community level (e.g., total biomass, community size structure, trophic level of the community) were most robust (i.e., showed the clearest trend) and that multiple indicators are necessary to identify fishing perturbations. These indicators could be used as performance indicators when compared to a baseline for management purposes. This study shows that ecosystem models can be valuable tools in identification of the system state in terms of complexity, stability, and resilience and, therefore, can complement biological metrics currently used by monitoring programs as indicators for coral reef status. Moreover, ecosystem models can improve our understanding of a system’s internal structure that can be used to support management in identification of approaches to reverse unfavorable states.

## Introduction

Resource managers are confronted with a range of challenges in their mission to sustain and restore coral reef goods and services that humans desire. Reductions in fishery harvests, whether a result of the degradation of fish habitat, following declines of target fish population, or increased regulation, will have substantial cultural, economic, and social implications for resource users. Effective management requires an understanding of coral reefs as ecosystems and of the complex and potential synergistic effects of different stressors [1], [2]. Globally, about three-quarters of all coral reefs are threatened by increased stress from pollution, extensive fishing, and climate change [3]. About half of the coral species that are very susceptible to bleaching are also heavily vulnerable to disease and predation, and recovery can be slow or absent [4]. At the Great Barrier Reef, coral cover has halved in the last three decades [5]. Ecological processes will interact with effects of global environmental change. For instance, herbivores (e.g., herbivorous fishes and sea urchins) can control the growth of algae and, therefore, facilitate coralline algal and coral settlement and growth, and they have been identified as a keystone group for their important role in structuring coral communities and improving reef resilience (i.e., the ability of a reef to absorb shock, resist phase shifts, and regenerate after natural and human-induced disturbances) [6]–[10]. Reductions in herbivorous fish biomass also may affect the microbial diversity with a shift to more pathogenic microbes and reduced microbial species richness, ultimately affecting the condition of the reef [11]. Areas protected from fishing or with less fishing pressure generally have higher live coral cover than do unprotected areas, and fish communities there have more large-bodied fishes [12], [13]. It is our opinion that management should focus on assessment and improvement of reef resilience to maximize the capacity of corals to respond to the imminent threats of global climate change [14], [15].

Resource managers and users can benefit from an evaluation of the system’s present status in terms of complexity, stability, and resilience–features that support biodiversity [16] and ecosystem *health* (health used in terms of high diversity, energy recycling, resilience) [17]. Fishing, habitat degradation, land-based sources of pollution, and global environmental changes all affect the health of coral reef ecosystems. Recently, coral reef models have been constructed to investigate ecosystem effects of fishing and alternative fishery management scenarios [18]–[22], habitat degradation [21], climate change [23], [24], and land-based pollution [25], [26]. Despite the increase in number of modeling studies in coral reef areas, there is still little information on the most appropriate indicators for changes in these systems [18]. Outcomes from ecosystem-based models can identify quantifiable metrics that reflect features of ecosystem’s structure and function, indicative for a system’s health under its level and type of perturbations [17], [27]. Establishing these indicators is among the first steps scientists can take to support the implementation of ecosystem-based management [2], [28]. Once these indicators are identified, the next step is to link them to criteria for management decisions; for example, indicator values X, Y, and Z that fall below a priori established threshold values will trigger a specified management action [29]. However, quantitative approaches for selection of ecosystem-level indicators are only beginning to emerge and, so far, mostly for pelagic systems [27], [30], [31]. These indicators might not be suitable for coral reef ecosystems because they differ in structure and energy flow (e.g., more complex food webs, including the microbial food web, for effective recycling of the limited nutrients in reef systems) and in fisheries (e.g., more diversified on reefs).

Empirical studies on coral reef structure and function have generally used spatial patterns or temporal trends in benthic cover and fish biomass and assemblages as indicators for perturbations to reefs from terrestrial runoff [32]–[34], climate change [35]–[37], and fisheries [e.g., 38,39–41]. These parameters are usually used as performance indicators for reef health in monitoring programs. However, they target only direct effects of fishing and do so mostly on small scales (e.g., fish biomass and size structure inside and outside MPAs) [38], [40], [42], [43], and no indicators exist for indirect ecosystem effects. Such indicators are crucial to an assessment of the overall ecosystem effects of target species removal and to allow holistic fisheries management [44]. Trophic mass-balanced models represent an analytical approach that could help evaluate ecosystem effects of fishing perturbations and identify optimal management scenarios [19], [30], [45]–[48].

**Figure 1:**
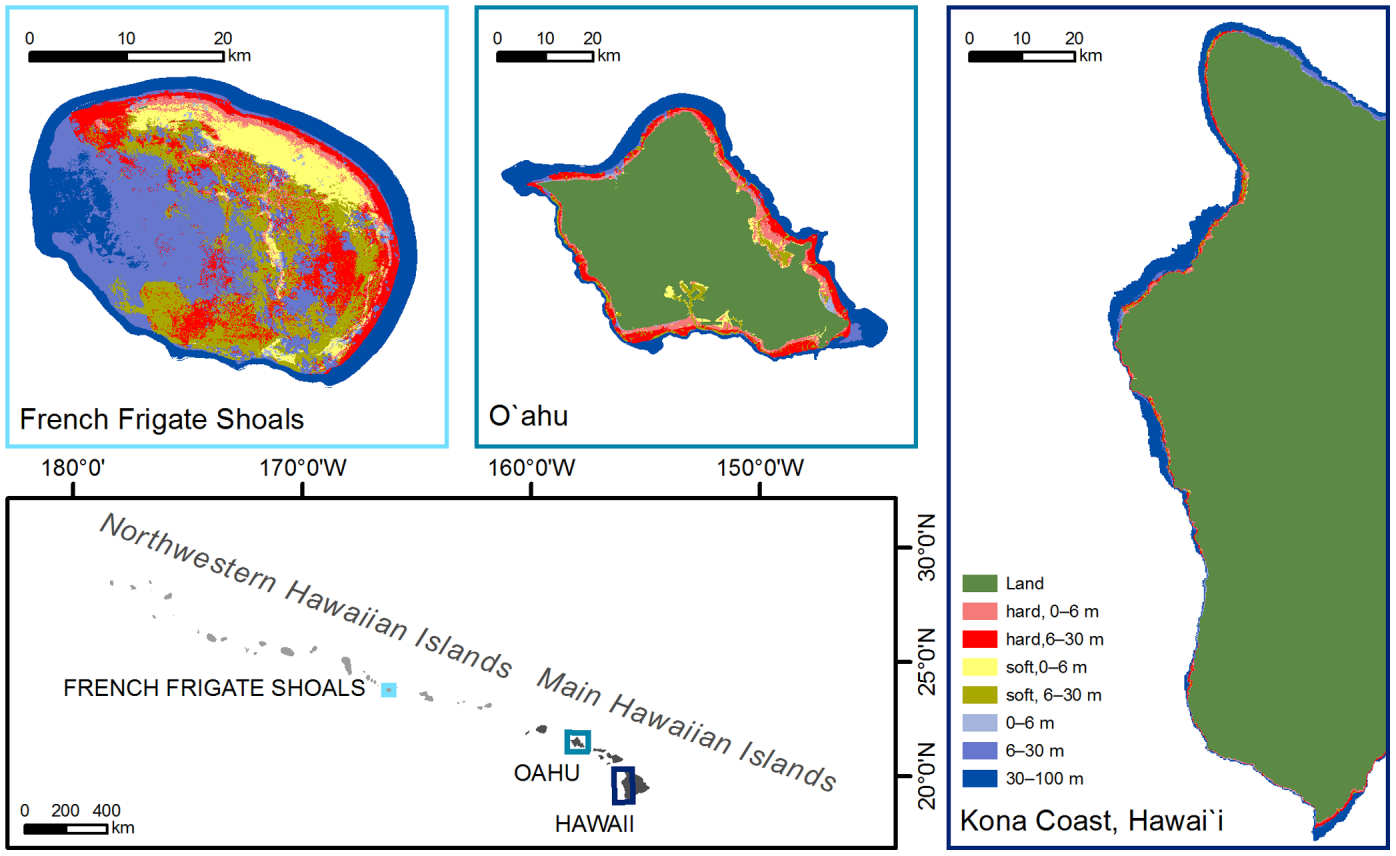
Habitat maps of the three modeled coral reef areas and their location in the Hawaiian Archipelago. Hard and soft in the legend indicate bottom type.

This study focuses on the quantitative description of the characteristics of ecosystem attributes of three coral reef systems along a fishing pressure gradient in Hawai’i, located in the middle of the Pacific Ocean (Fig. 1). We attempt to identify the most reliable indicators of ecosystem structure and function of coral reefs to support ecosystem-based fishery management. This comparative approach along an exploitation gradient is used to identify a range of indicators against which each system is assessed in relative terms. The model used is validated with empirical assessments from field data, and the suitability of performance indicators presently used for coral reef management in Hawai’i is discussed.

## Materials and Methods

### Study Sites

We selected three systems along a gradient of fishing pressure on the basis of human population and commercial catch statistics (www.pifsc.noaa.gov/wpacfin/hi/dar/Pages/hi_data_3.php. Accessed 2011 Jan): (1) French Frigate Shoals (FFS) in the Northwestern Hawaiian Islands–no fishing, (2) Kona Coast of Hawai’i Island–medium fishing, and (3) O’ahu–heavy fishing (Fig. 1, Table 1).

**Table 1:** Characteristics of the three coral reef areas included in this study.

Reef system	Lat.	Long.	0–30 m area(km^2^)	% Hard-bottom habitat	Humanpopulation^1^	Population/km^2^ reef	Exploitation (% of total state catch)
French Frigate Shoals	−166.21	23.79	163	54	0	0	0
Kona	−155.42	19.53	90	76	47,705	530	5
O’ahu	−158.00	21.49	423	72	953,207	2,253	50

1 US Census Bureau 2010 estimate.

Exploitation indicates fishery exploitation.

Models represent the status of the shallow-water (<30 m), hard-bottom, forereef ecosystems and are based largely on 2010 data. Total forereef area is 88 km^2^ around FFS, 68 km^2^ along the Kona Coast, and 307 km^2^ around O’ahu (NOAA Fisheries Coral Reef Ecosystem Division (CRED) unpubl. data). The monthly mean sea-surface temperatures vary between 24°C in winter and 27°C in summer [49]. Situated in the middle of the North Pacific Ocean, the reefs are exposed to large winter swells that pound on the coastline from the northwest, summer swells from the south, and strong trade winds from the northeast. Hawai’i is located in the North Pacific Subtropical Gyre, which is characterized by low upwelling [50] and low plankton standing stock [51]. Climatologic chlorophyll-*a* standing stock from the open ocean were similar between the three areas with annual averages between 2004 and 2010 of 0.057 mg/m^3^ (SE 0.003) for Hawai’i and 0.066 mg/m^3^ (SE 0.004) and 0.067 mg/m^3^ (SE 0.005) for O’ahu and FFS, respectively (CRED unpubl. data).

### Data

This study used data on coral reef fish assemblages, benthic cover, invertebrate assemblages, insular microbe and phytoplankton biomass all collected with the same suite of methods for each study site by the NOAA Pacific Islands Fisheries Science Center (PIFSC) as part of the Pacific Reef Assessment and Monitoring Program (Pacific RAMP). Benthic and fish surveys were conducted between 2001 and 2010 using Rapid Ecological Assessment (REA) surveys at long-term sites. In the earlier years (2001–2007) belt-transect surveys were conducted at fixed mid-depth (12–15 m) forereef sites. Since 2007, for Pacific RAMP, PIFSC implemented a stratified random survey design in forereef, hard-bottom habitats <30 m using belt-transect visual surveys for benthic cover and invertebrates and stationary-point-count (SPC) visual surveys for fish data (for details on SPC surveys, see [39]). Fish length estimates from visual censuses were converted to weight using the allometric length-weight formula: W = *a*TL*^b^*, where parameters *a* and *b* are constants, TL is total length in millimeters, and *W* is weight in grams.

Length-weight fitting parameters were available for 150 species (68% of all species included in the model) commonly observed on visual fish transects in Hawai’i (Hawai’i Cooperative Fishery Research Unit unpubl. data). These data were supplemented with information from other published sources and from studies reported on FishBase (www.fishbase.org) that were conducted in other tropical regions on the same species. The Kona Coast model also included fish and echinoid data collected using belt-transect surveys between 2002 and 2010 on mid-depth forereef habitats by the Division of Aquatic Resources (DAR). Towed-diver survey results for roving predatory fishes were used for all three models because that method appears most suitable for fishes that are highly mobile and heavily clumped or for rare fishes [52]. Echinoderms often have a patchy distribution, and data from towed-diver surveys that cover a large area (∼ 2000 m^2^ vs. ∼ 50 m^2^ for Rapid Ecological Assessment (REA) surveys) are likely more accurate for conspicuous species (e.g., crown-of-thorns sea stars, large urchins, sea cucumbers). However, for boring urchins, it is difficult to obtain a reliable count by towed divers; therefore, we used a combination of belt-transect and towed-diver surveys for echinoderms. Phytoplankton and microbe data were derived from water samples taken at the surface and at ∼ 1 m above the reef. Phytoplankton biomass was calculated from the chlorophyll-*a* concentration measured in the water samples, and insular bacteria biomass was calculated from the counted numbers of cells per milliliter. Ratios of production over biomass (P/B) and consumption over biomass (Q/B) came from published sources or empirical relationships following Pauly [53] and Palomares and Pauly [54] for fish and Brey [55] for nonfish groups. The supporting information gives details on the input parameters of all functional groups and includes the diet composition matrices (Text S1, Tables S1, S2, S3, S4, S5, S6, S7, S8).

### Model

We constructed a mass-balance ecosystem model using the Ecopath with Ecosim v.6 software (www.ecopath.org). Ecopath is a steady-state mass-balanced model, determined largely by trophic interactions and fishery removals, can be used to describe and examine the energy flows in ecosystems, and provides insight into ecosystem maturity and functioning [56]. Ecopath was first developed by Polovina [58] and further advanced by Christensen and Pauly [57]. This modeling approach is based on a set of simultaneous linear equations for each functional group (state variable) in the system, where the production of a given group is equal to the sum of all predation, nonpredatory losses, and exports [56], [58], [59]. Each functional group in the model is represented by one balanced equation and requires five input parameters. Export and diet composition of each group are mandatory, and three of the four parameters–biomass (B), P/B, Q/B, and ecotrophic efficiency (EE)–also must be entered for each group. The linear equations are then solved and the unknown parameters are estimated. The most robust approach is to enter B, P/B, and Q/B and allow the model to estimate EE. This approach also provides a check for the mass balance because EE cannot be greater than 1.

We included in the model 33 functional groups representing 2 detritus groups (detritus and carrion), 6 microbial food web groups (phytoplankton, 2 groups of bacteria, 3 groups of zooplankton), 3 benthic primary producers, 9 invertebrate groups, 11 fish groups, 1 marine reptile, and 1 marine mammal group (Text S1). Species were aggregated into those groups on the basis of similarities in habitat use, diet, feeding behavior (i.e., roving, hunting, grazing), life-history characteristics (e.g., max age, growth constant, length at first maturity), and ecological role (i.e., excavators or bioeroders, scrapers, grazers or detritivores, browsers). Because of their potentially important ecosystem roles and impacts, sea urchins (key herbivores) and sea stars (coral predator) were included as distinct functional groups.

We added constraints on the EE, to range between 0 and 0.95, and used the default value for the assimilation efficiency of 80% for all groups. About 80% of the consumption was assumed to be physiologically useful for consumer groups, and the nonassimilated food (20%, consisting of urine and feces) was directed to detritus [57]. However, that default value tends to underestimate egestion by herbivores and detritivores. Thus, assimilation efficiency was adjusted to 70%, for herbivorous fish groups to 70% for demersal and carnivorous zooplankton, and to 60% for bacteria, herbivorous zooplankton, and benthic deposit feeders [56], [60]–[62].

To achieve mass-balance in the model, we modified the diet data slightly because these data were the most uncertain parts of the four main input values (B, P/B, Q/B, and diet). After mass-balancing, the trophic level for each functional group was calculated by the model as were various network flow indices that measure the ecosystem maturity following Odum [63] and Ulanowicz [64]. The Kona Coast model showed EE was greater than unity for some invertebrate groups, indicating that the lower trophic levels had insufficient biomass or production to support the consumption of the higher trophic levels. To address this problem, the EE was set to the default value 0.95 to allow Ecopath to calculate the biomass. This approach is considered valid because this Ecopath model is a top-down model and scales the flows to the food required to maintain the biomass at the top of the food web [65], [66], and we are confident in the comparison of our estimates of the biomass for these higher trophic levels between the three models because they were all obtained through the same visual survey methods. Plankton biomass needed to be increased for the FFS and Kona models to ascertain enough biomass to sustain the total consumption. In coral reef systems, phytoplankton grazing is a principal pathway that allows allothonous nutrients [67] and suspended particulate matter [68] to import to a reef community through the flowing water. Feeding rates increase when water flows over the reef [67], [69], [70], and the shape of the benthic community structure on a reef developed by the currents and waves increases capture efficiencies [71], [72]. Therefore, it is believed an increase in plankton biomass from flows over the reef is valid [73].

Validation of the model structure was conducted through comparison of Ecopath’s pedigree index with other Ecopath models. Ecopath estimated the pedigree index, on the basis of the confidence intervals (CI) of each input parameter, which describes how well rooted the model is in local data on a scale of 0 to 1, with 1 being the best [74]. Confidence in data from field sampling was assumed to have the narrowest CI (10%–30%), and estimates from other models or calculated by Ecopath were assumed to have the widest CI (40%–60%). Most of the biomass data were obtained from Pacific RAMP field surveys and other published field studies from Hawai’i. Therefore, they were defined as having a 10%–30% CI of the mean; whereas, P/B and Q/B input parameters were defined as having 20%–60% CI, depending on whether they came from field studies (∼ 20%), empirical relationships (∼ 40%), or other models (∼ 60%). Diet data (from literature and Fishbase) were defined as having 40% CI when it came from qualitative studies in Hawai’i, 50% from expert opinion, and 80% from quantitative studies. Fishery data were assigned a 50% CI.

Sensitivity analyses were conducted using manual substitution of values (+25%, +50%, –25%, –50% of original number) for biomass, P/B, and Q/B for cryptic or small invertebrate groups, because these were the groups with the most limited survey data, and examination of the effect of these changes on the basic input parameters.

### Fishery

We defined two fishery “fleets”: recreational and commercial. Commercial fishery data were compiled from records of the State of Hawai’i commercial fish landings using the NOAA PIFSC’s Fishing Ecosystem Analysis Tool (FEAT; www.pifsc.noaa.gov/human_dimensions/fishing_ecosystem_analysis_tool.php. Accessed 2011 Jan), a geospatial tool that summarizes commercial fisheries landing statistics per species and fishery region. These fishery data include coastal and pelagic fisheries. The Ecopath models in this study were limited to the shallow (0–30 m) reef areas with fish biomass estimated only from this area. We assumed that the coastal fishery data captured the extraction of top predators in the modeled area sufficiently, and, therefore, we excluded the pelagic fishery data. We also included landings from the aquarium trade in the commercial fishery fleet using data from Walsh et al. [75]. The aquarium trade is concentrated on the Kona Coast of Hawai’i, where 75% of the total state reported landings originate; therefore, this fishery is included only in the Kona Coast model. Recreational catch data came from the DAR Hawai’i Marine Recreational Fishery Statistics program (www.st.nmfs.noaa.gov/st1/recreational/index.html. Accessed 2011 Jan). Again, we excluded pelagic species. We compared the results of the recreational fishery with published creel surveys conducted in Hanalei, Kaua’i, Kane’ohe Bay, O’ahu [76], [77], and Puako, Hawai’i (J. Giddens pers. comm. October 2011). Because of the large discrepancy between results from creel surveys and the reported commercial and recreational landings, we calculated “correction” factors using these values for some fish groups (Text S1).

To calculate the fishing mortality, we divided the yield (t/km^2^/y) by the estimated standing stock per functional group. The standing stock estimates used the Pacific RAMP daytime visual surveys. Because these surveys omit cryptic and nighttime species, values likely underestimate actual stock size. However, yield likely is underestimated as well because the nighttime fishery is not accounted for in the recreational landings or creel surveys; therefore, we believe that estimated fishing mortalities are still conservative estimates. Recreational fishery is reported for the entire state. For this fishery, we assumed the same proportion of statewide landings to landings per fishery region as retrieved from the FEAT model for commercial landings of reef fish. In other words, 50% of the total reef fish landings were from O’ahu and 5% from Kona.

### Candidate Indicators for Ecosystem Status under Fisheries Exploitation

We selected a suite of candidate indicators for ecosystem structure and network flows (Table 2) based mostly on the robust indicators identified by Fulton et al. [30], who evaluated 31 ecological indicators with potential to detect effects of fishing between aggregation levels, two model types, and four fishing pressure scenarios. We supplemented those indicators with reliable indicators identified by Arias et al. [18], Samhouri et al. [31], Shin et al. [28], and Xu et al. [46] and with indicators used by the State of Hawai’i for coral reef monitoring. We used the following criteria to select ecosystem indicators: (1) indicators reflect well-defined ecological processes occurring under fishing pressure, (2) trends in the indicators are expected to be closely correlated with trends in fishing pressure; (3) indicators are easily measurable or estimated in monitoring programs. Included in Table 2 are criteria for mature and, in general, more resilient systems.

**Table 2:** Selected candidate indicators for coral reef ecosystem effects of fishery.

#	Candidate Indicator	Explanation	Expectation with increased fishery exploitation
**1**	Net primary production (NPP)	Activity index for lower trophic levels.	increase (zero for mature ecosystems)
**2**	Net system production	Sum of biomass accumulation, biomass lost to mortality, andbiomass lost to migration of all benthic species.	Increase (close to zero for mature systems)
**3**	Total Biomass (B)	Sum of biomass for all ecosystem species.	decrease
**4**	B - sharks and jacks	Biomass of apex predators.	decrease
**5**	B - planktivores	Biomass of planktivorous fish.	increase
**6**	B/P – size structure	Biomass to productivity ratio as an indication of the sizestructure of the organisms in the system.	Decrease (higher value indicates more mature system)
**7**	Piscivores:planktivores biomassratio	Biomass ratio of piscivorous and planktivorous fish groups.	decrease
**8**	Total catch	The biomass of functional groups targeted by fisheries.	increase
**9**	Trophic level of catch	Biomass-weighted average of trophic level of allspecies caught.	decrease
**10**	Fishery gross efficiency	Indicates the importance of fishery in structuring the systemstructure (0.00002 is global average).	increase
**11**	Mean trophic level of community	Biomass-weighted average trophic level of all species inthe ecosystem.	decrease (higher value indicates more mature system)
**12**	Total consumption	The sum of somatic and gonadal growth, metabolic costs,and waste products for all modeled species.	decrease (higher value indicates more mature system)
**13**	Total respiration	The portion of consumed energy that is not used forproduction or recycled as metabolic waste indicative for the systems activityof the higher trophic levels.	decrease (higher value indicates more mature system)
**14**	System’s omnivory index (SOI)	The variance of the trophic level of a consumer’s prey group(i.e., specialist, such as coralivorous fish, vs. generalist,such as omnivorous hermit crabs). This indexcharacterizes the extent to which a system displaysweb-like features.	decrease
**15**	Ratio of primary production torespiration (PP/R)	The ratio of total production relative to total respiration.	increase (one for mature ecosystems)
**16**	Primary production required (PPR) forsustaining fish biomass consumption	Calculated primary production required by the system tosustain the level of fishery.	increase
**17**	Finn’s mean path length	The average number of functional groups that a unit ofenergy flows through in the system before being lost(food chain length).	decrease (higher value indicates more mature system)
**18**	Finn’s cycling index	The fraction of all flows in the ecosystem that is recycled.	decrease (higher value indicates more mature system)
**19**	Predator cycling index	The fraction of all flows in the ecosystem recycled throughnon-detrital pathways indicates the importance of predationin the structure and functioning of the system at highertrophic levels.	decrease
**20**	Total system throughput (TST)	Represents all of the biomass flows and is the summationof consumption, respiration, export and flows to detritus.	decrease (higher value indicates more mature system)
**21**	Capacity	Measurement of size and complexity of the system,calculated as the product of TST and the maximum degreeof specialization.	decrease (higher value indicates more mature system)

These indicators were selected from literature reviews and a brief description (explanation) and expected response to fishery is given.

## Results

### Model Structure and Sensitivity

The Hawai’i Ecopath models’ pedigree index values were 0.50 for FFS, 0.59 for O’ahu and 0.62 for Kona; all values fell in the medium–high range compared to 50 other Ecopath models, 48% of which had a pedigree from 0.40 to 0.59 and only 10% of which had a pedigree higher than 0.60 [78]. These results suggest that the model is well rooted in local data and, therefore, robust.

**Figure 2:**
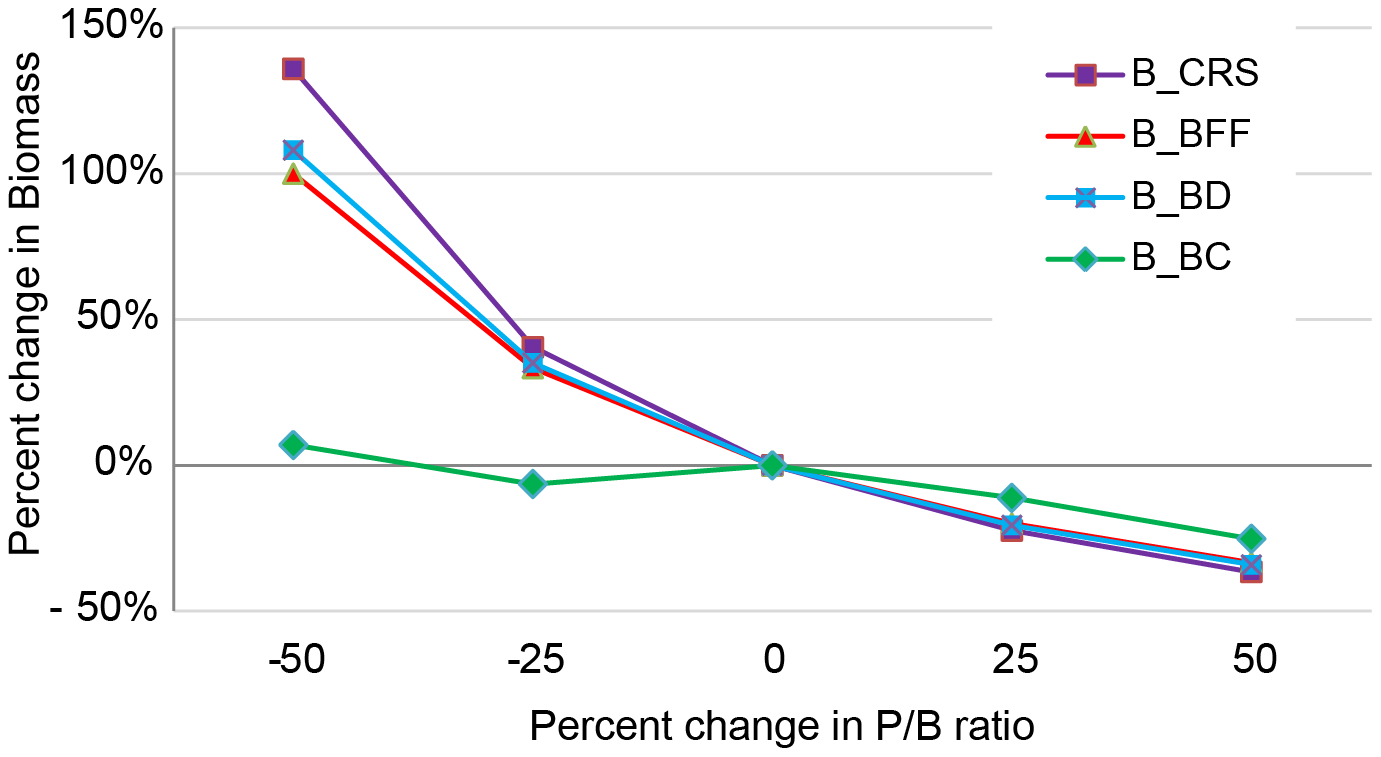
Results of sensitivity analysis of four invertebrate groups to changing the P/B ratio on the biomass.

Sensitivity analyses showed that the model was least sensitive to a change in Q/B ratio for the meiobenthos (e.g., benthic filter feeders, benthic carnivores, benthic deposit feeders and crustaceans), with only crustacean biomass changing more than 10% with a 50% applied increase or decrease of their Q/B ratio. However, decreasing the Q/B by 50% resulted in an unrealistically high ratio of production over consumption (P/Q) of >1 for benthic filter feeders. Benthic carnivores were the least sensitive group to changes in P/B ratio compared to the other small invertebrate groups (Fig. 2). Exploration of the sensitivity of the Q/B ratio with a decreasing biomass or P/B ratio (–25% and –50%) resulted in failure of the Ecopath model to calculate the EE. In comparison, elevation of these values resulted in a very high increase in the Q/B ratio, especially for benthic filter feeders as a response to a biomass increase and for benthic detritivores as a response to a P/B ratio increase. P/Q ratio values were unrealistically low (<0.05) for all groups when biomass was changed and for all groups except the benthic carnivores when P/B changed. In contrast, biomass and the P/B ratio were not very sensitive to increasing P/B ratio or biomass, respectively, but more so to decreasing those values except for the biomass of benthic carnivores. Clearly, more study needs to be devoted to these invertebrate groups to obtain a better estimate of their biomass and P/B ratio for model improvement.

### General Description of the Three Systems

**Figure 3:**
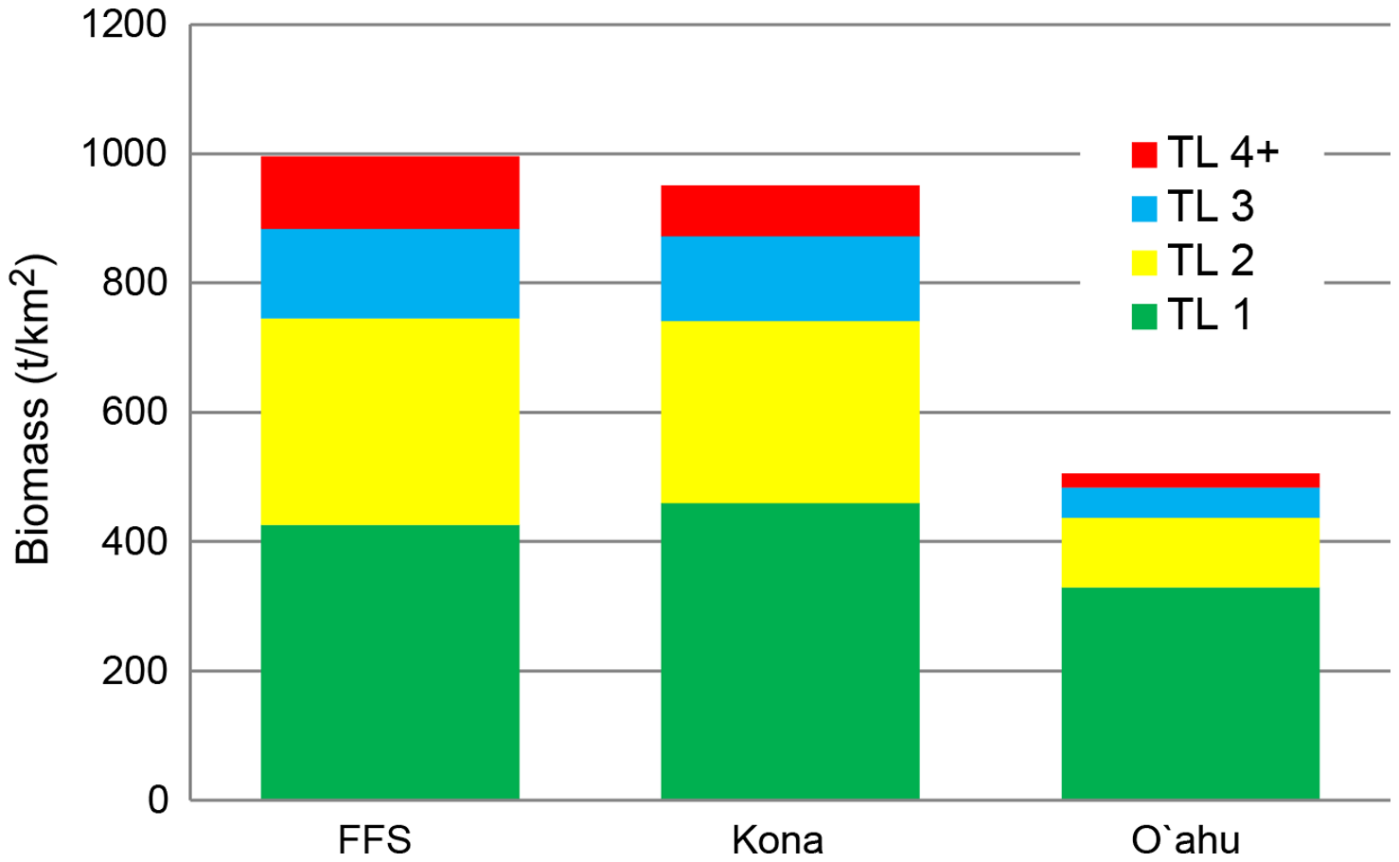
Composition of biomass (t/km^2^) per trophic level (TL) for the three systems studied in Hawai’i. FFS is French Frigate Shoals; Kona represents the Kona Coast of Big Island.

Ecopath aggregates an entire system into distinct trophic levels sensu Lindeman [56]. FFS showed a higher overall biomass with the main differences in the higher trophic groups (Fig. 3). The models estimated that the majority (57%–64%) of the energy flows originated from detritus rather than from primary productivity, indicating that secondary production is based mainly on detritus and net primary production enters the coral reef food chain through heterotrophic benthic organisms. Transfer efficiency was highest from trophic level I to II, especially for the energy flow from detritus, suggesting high energy efficiency at the lower trophic levels. Although the total biomass values for the Kona system and the FFS system were similar, the transfer efficiency for the higher trophic levels (5 and up) was 1.5 to 2.5 times higher in FFS compared to both O’ahu and Kona. The importance of detritus and high efficiency in recycling also was corroborated by the high values for Finn’s cycling index, especially in the models with no (FFS) or intermediate (Kona) fishing perturbation.

### Evaluation of Indicators based on Analyses of Survey Data

Benthic indicators derived by field surveys did not show any clear relationship with fishing pressure (Table 3); thus, habitat parameters alone cannot be used as fishery indicators. However, fish indicators did reflect the fishing pressure gradient. Direct effects of fishing were reflected in the increase in total catch and decrease in biomass of apex predators (roving piscivores and sharks) and of large-sized (≥50 cm) fishes with increasing fishing pressure (Table 3). Also, total fish biomass showed high values at FFS, intermediate values at Kona, and low values at the most populated (highest fishing pressure) island of O’ahu. The disparity in biomass of large fishes and apex predators between FFS and Kona is noteworthy in that it is much greater than the disparity between Kona and O’ahu, indicating that these indicators are quite crude and that the effect of fishing is almost binary (populated/unpopulated).

**Table 3:** Benthic (B) and fish (F) related indicators for coral reef health from survey data (unnumbered; NOAA Fisheries Coral Reef Ecosystem Division and Hawai’i Department of Aquatic Resources indicators) and candidate indicators (numbered) for fishery effects.

No	B/F	(Candidate) Indicators	FFS	Kona	O’ahu
	B	Total biomass benthic algae (g/m^2^)	281	225	307
	B	Total cover macroalgae (%)	12.5 (6.44)	2.3 (0.92)	17.7 (2.24)
	B	Total cover crustose coralline algae (%)	8.0 (5.01)	8.9 (0.94)	6.8 (0.88)
	B	Coral cover (%)	20.3 (6.61)	24.6	11.3 (1.36)
	B	Habitat complexity (towed-diver surveys 2008–2010; 1 is low, 5 is high)	2.2	2.9	1.9
	F	Total fish biomass (Rapid Ecosystem Assessment surveys 2005–2010) (g/m^2^)	92	68	20
	F	Large (≥50 cm) fish biomass (towed-diver surveys 2006–2010) (g/m^2^)	6.9	1.4	0.8
**4**	F	Biomass apex predators (sharks and roving piscivores) (g/m^2^)	4.86	0.30	0.26
**5**	F	Biomass planktivores (g/m^2^)	19.09	12.94	4.50
**6**	F	Piscivores:planktivores biomass ratio (g/m^2^)	0.33	0.52	0.23

The numbers correspond to the numbers in Table 2 for details on these indicators. Standard error given in parenthesis. FFS is French Frigate Shoals; Kona is the Kona Coast of Big Island.

Against expectations, results show that biomass of planktivores (e.g., *Melichthys niger*, *Naso hexacanthus*, *Myripristis* sp., *Chromis* sp.) strongly declined with an increase in fishing pressure (Table 3). Planktivorous fishes are mostly prey fishes, and their biomass is expected to go up with a release of predation pressure [30].

### Evaluation of the Candidate Indicators on the Basis of Ecosystem Structure and Network Analyses

Various candidate indicators showed a strong trend with increasing fishing pressure (Tables 3 and 4). Sequential ecosystem structure effects along the fishing pressure gradient were most clearly reflected by fishery-related indicators, net system production, size structure of the community, and biomass of planktivores (Tables 3 and 4). The relatively high fishery gross efficiency for O’ahu suggests that that system structure is strongly influenced by fishing. The negative value for the system production at FFS indicates large import. Import is expected to be much higher at the forereef habitat of FFS because it is adjacent to a large lagoonal area, compared with the steep drop-off at the Kona Coast and the limited, shallow, lagoonal bays around O’ahu (Fig. 1). In our ecosystem network analyses, similar clear patterns were shown by Finn’s mean path length, Finn’s cycling index, and the primary production required to sustain the fishery (Table 4).

**Table 4:** Ecopath derived values for candidate indicators of fishery effects on coral reef ecosystems.

No.	Candidate Indicators	FFS	Kona	O’ahu	units
**1**	Net primary production (NPP)	7,057	8,739	6,403	t/km^2^
**2**	Net system production	−158	517	3175	t/km^2^
**3**	Total Biomass (B) exl. detritus	996	951	539	t/km^2^
**7**	B/P – size structure	0.069	0.061	0.057	
**8**	Total catch	–	0.76	1.31	t/km^2^/y
**9**	Mean trophic level of catch	–	2.96	3.11	
**10**	Fishery gross efficiency	–	0.000087	0.000205	
**11**	Mean trophic level of community	1.93	1.82	1.54	
**12**	Total consumption	21,056	21,715	9,187	t/km^2^
**13**	Total respiration	7,215	8,223	3,228	t/km^2^
**14**	System’s omnivory index (SOI)	0.291	0.236	0.241	
**15**	Ratio of primary production to respiration (PP/R)	0.98	1.06	1.98	
**16**	Primary production required (PPR) to sustain fishery	0	26	142	t/km^2^
**17**	Finn’s mean path length (Food chain length)	5.11	4.45	3.57	
**18**	Finn’s cycling index	28.42	22.92	16.01	% of TST
**19**	Predator cycling index	3.97	4.01	3.75	% of TST w/o detritus
**20**	Total system throughput (TST)	37,817	40,352	23,493	t/km^2^
**21**	Capacity	207,484	226,151	119,837	flowbits

The numbers correspond to the numbers in Table 2 for details on these indicators. FFS is French Frigate Shoals; Kona is the Kona Coast of Big Island.

The remaining candidate indicators did not show a clear sequential pattern with increases in fishing pressure, but many indicators pertaining to the system’s stability or maturity sensu Odum [63] showed a binary pattern, with a minimal difference between FFS and Kona (Table 4). For example, mature, stable systems have a close coupling between production and respiration (P/R ∼ 1) and, therefore, have no or little excess production, a high system throughput and capacity, and high overall biomass. Other indicators that showed the same binary pattern were total biomass, mean trophic level of the community, and biomass of roving piscivores. On the basis of the indicators for system maturity (Table 4), it appears that the reef system around O’ahu is in a more transitional state compared to the reef systems around FFS and along the Kona Coast. This difference could be a result of higher fishing perturbations as habitat (benthic indicators) did not show this trend.

Candidate indicators that did not show a simple linear pattern with an increase in fishing pressure were the piscivore:planktivore ratio and the net primary production. The system’s omnivory index showed minimal to no differences among the three systems, indicating that the complexity of the food webs was similar. The trophic level of the catch was also similar between Kona and O’ahu.

## Discussion

The results should be regarded as trends as it is impossible to make rigorous statements on the basis of only three points. Ideally, more Ecopath models will be developed for other islands in the Hawaiian Archipelago to get a better understanding of which combination of variables are most indicative for fishing pressure.

### Model Structure and Sensitivity

In coral reefs, roughly 50% of the net primary production (NPP) produced offshore and on the reefs is channeled through the microbial loop [79]–[81]. This high efficiency in reefs was successfully simulated in the model on the basis of the high detritus dependence and the high value of Finn’s cycling index, especially for the Kona and FFS models. Including the microbial food web in the model increased total energy throughput and energy transfer efficiency (TE) from detritus but decreased the TE from primary productivity (PP). These effects could be caused by enhanced recycling of materials and energy by the microbes; therefore, including the microbial loop simulates the system behavior more appropriately [82]. In all three models, TE was 1.4 to 1.8 times higher from detritus than from primary production, corroborating the importance of the microbial loop in coral reef ecosystems. On the basis of the pedigree, it was clear that all three models are highly rooted in local data enhancing the robustness.

Area is an important variable that influences model results. Comparison of our model results with results from other regional models was difficult because study area, survey methods, and functional groups varied between models. The FFS Ecopath model of Parrish et al. [83] also has a shallow (0–30 m) reef component, and, when comparable areas were derived, the fish biomass in Parrish et al’s model was 94.3 g/m^2^, which compared very well with our 91.6 g/m^2^. It was not possible to compare any other functional groups. A Kona coast model (Wabnitz unpubl. data) includes the same shallow reef area that was used in this study but also extends to a depth of 100 m and includes all habitat types for a total study area of 90 km^2.^ In our study, we only used the forereef area at depths of 0–30 m for a total area of 68 km^2^. Wabnitz (unpubl. data) used shallow (0–30 m) fish biomass values from Friendlander et al [84], and our estimate of 67.7 t/km^2^ for our Kona coast model is very comparable with their hard-bottom estimates (ranging between 40 and 85 t/km^2^) for their four Kona sites. We feel, therefore, confident that our fish biomass numbers are realistic.

The lower trophic groups have been considered mostly as biomass pools in other reported reef models and are the groups of greatest uncertainty; hence, variation can be expected. Urchin biomass in this study was 19 t/km^2^, which was 5 times lower than values from Wabnitz (unpubl. data). Another discrepancy between Wabnitz (unpubl. data) and this study was the biomass of corals. Coral biomass in this study adjusted for the sand habitat (no corals) was 194 t/km^2^ and in Wabnitz et al 82 t/km^2^. This large difference in coral biomass could be caused by the (assumingly) low coral cover in the mesophotic depth included in the Wabnitz (unpubl. data) study area. Clearly, more research on invertebrates would greatly enhance the model. Sensitivity analyses of the meiobenthos showed that changes in the Q/B ratio had little effect on the biomass or P/B ratio, but decreasing the biomass by 50% resulted in a change in P/B of 80%–100% for all four invertebrate groups and decreasing the P/B ratio resulted in a change in biomass of 100% for benthic filter feeders and deposit feeders and 136% for crustaceans. Our biomass estimates for these groups came from studies of the Kona Coast of Hawai’i supplemented by visual observations at hard-bottom sites in each system, and the P/B (and Q/B) ratios were weighted according to the species composition at each system and are in the range of values reported in other reef systems (Text S1). Because these lower trophic functional groups play an important role in the transfer efficiency of energy, better estimates are highly recommended to improve the model.

### Evaluation of Indicators Derived by Monitoring Programs

Coral and macroalgal cover are variables that are widely used as metrics in evaluating reef health and are also included in DAR’s monitoring program and the Pacific RAMP. Solely on the basis of these habitat indicators, reefs along the Kona Coast of Hawai’i (intermediate fishing) would be categorized as being in a better health than are reefs in FFS (no fishing; Table 3). Therefore, these variables are not directly indicative for fishery effects. Fishing does not necessarily degrade reefs, high macroalgal cover does not necessarily indicate a degraded reef [85], [86], and high coral cover does not necessarily indicate a reef with high fishable biomass [87].

In contrast, large-fish biomass and the candidate indicator, biomass of apex predators, showed a strong relation with fishing pressure; albeit, not a sequential relation, it was more a binary pattern where intermediate fishing pressure resulted in a sharp decline in biomass of these species.

The piscivore:planktivore ratio was one of the indicators that was most robust in other system studies [30] but was not an effective indicator of fishing pressure on the Hawaiian reef systems. This result could be because the biomass estimates for apex predators from towed-diver surveys were used. If biomass estimates from REA (small-scale) surveys were used, the piscivore:planktivore ratio would be 3.34 for FFS, 0.52 for Kona, and 0.19 for O’ahu, where shark and jack encounters in REA surveys are rare. This deceasing trend is what you would expect along a gradient of increasing fishing mortality with target species declining and planktivorous species contributing a larger part to fish assemblages [30]. However, in fished areas, jacks and sharks are likely wary of divers and swim away, and their numbers can be underestimated; whereas, in protected areas (such as FFS), jacks and sharks might be more curious and approach divers, hence, their numbers are likely to be overestimated at REA sites. Therefore, the biomass estimates from towed-diver surveys are believed to be more accurate [52].

The same holds true for the biomass of planktivores, which, against expectations, decreased with increasing fishing pressure. This phenomenon could be explained by the presence of a second food web driven by primary production (i.e., plankton–planktivorous fish–apex predators) that is intertwined with the detritus food web. The high EE for planktivores in FFS (0.947 vs. 0.680 in Kona and 0.425 in O’ahu) supports that theory. Another possible explanation is fishing mortality; according to the Hawai’i fishery statistics, soldierfishes (*Myripristis* sp.), unicorn fish (*Naso breviosis*), and some sergeant fishes (*Abudefduf* sp.) are targeted in the fishery and could drive their numbers down in the populated areas. Although the exact drivers of this phenomenon are unknown, this trend of high planktivorous biomass in remote areas compared to populated areas also is observed elsewhere in the Pacific [39].

### Evaluation of Candidate Indicators Derived by Ecopath

Evaluation of the candidate indicators across a fishing pressure gradient showed that indicators at the community level were most robust (i.e., clearest trend) and that multiple indicators are necessary to identify fishing perturbation. Candidate indicators related to the system’s community, such as total biomass, community size structure, and trophic level of the community, were indicative of fishing pressure and could be used as performance indicators compared to a baseline (e.g., a 1950 system).

Community and ecosystem attributes deal with energy flows and ecosystem functioning and are not readily measurable from field studies. Throughput, production, and consumption–along with the internal state (i.e., Finn’s cycling index, mean path length)–reflect a system’s ability to support its current state and level of exploitation in the long term [30], [88]. On the basis of the statistics of metrics indicative of a system’s maturity sensu Odum (1969), reefs around FFS and along the Kona Coast were in a stable, mature situation and the observed level of fishing along the Kona Coast was supported by the system. However, the coral reef ecosystem around O’ahu appears to be in a transitional state and fishing mortality is assumed to have played an important role in the current structure and functioning of this ecosystem. The mean trophic level of the community was an indicator that showed a clear decreasing trend with an increase in fishing pressure across the three study areas as did the biomass/production ratio, the mean food chain length, and Finn’s cycling index. The low EE for benthic algae (0.16–0.33) indicates a lack of herbivorous grazing pressure that could drive the transitional state (i.e., moving from a coral dominated system to an algal dominated system). Results from 7 out of 10 long-term (>10 year) monitoring programs in O’ahu showed a coral cover decline of 4% to 35%, corroborating this hypothesis [49]. Naturally, fishing mortality is not the only perturbation that affects the status of coral reef ecosystems. We used human population as an indicator for fishing intensity; however, with an increased population, other stressors to reef ecosystems, such as sedimentation, nitrification, and other land-based sources of pollution, also augment. Notwithstanding, the results from the Ecopath models in our study do show that fishing-related indicators did indicate that a clear decreasing trend with an increase in fishing mortality and benthic indicators did not.

### Conclusions

Candidate indicators for fishing pressure showed that indicators at the community level (e.g., total biomass, community size structure, trophic level of the community) showed the clearest trend with increased fishing mortality. Results also showed that multiple indicators are necessary to identify fishing perturbations. These indicators could be used as performance indicators when compared to a baseline for management purposes. Currently, collected data from monitoring programs in Hawai’i of fish biomass and fish assemblages and size structure clearly show a strong relation to fishing mortality, with higher fishing mortalities resulting in a shift in fish communities (decrease in number of large fishes and in biomass of piscivores), unlike data of benthic parameters (e.g., coral or algal cover). Ecopath statistics of the structure and functioning of ecosystems can supplement these metrics with insights into the stability of the system. Stable, mature systems are more likely to recover from perturbations, such as global change or local stressors to reefs (e.g., land-based sources of pollution, fishing). Understanding the processes that structure a reef is important in supporting marine resource managers to reverse transitional states to stable systems that yield high fishable biomass. On the basis of the results of this study, it is clear that the reefs around O’ahu are in a transitional state. The low EE for benthic algae around O’ahu, compared to around Kona and FFS, indicates that grazing pressure was minimal. Reduced grazing of (especially) macroalgae by herbivores could result in a shift to a system that is dominated by algae instead of corals; the latter is economically and aesthetically more desirable as it supports a higher fishable biomass and dive tourism. In follow-up studies, it would be beneficial to use Ecosim, a simulation model that uses Ecopath for input parameters, to evaluate management scenarios that are most likely to succeed in reversing the current transitional state of the coral reefs around O’ahu.

## Supporting Information

Figure S1 Schematic representation of the marine microbial loop. DOM is dissolved organic matter; EOC is extracellular organic carbon; DFAA is dissolved free amino acids; HNF is heterotrophic nanoflagellates or protists. Diagram created by Tracy McDole, San Diego State University.
(TIF)

Table S1 The weighting factors in percentage and input values with their source for the production over biomass (P/B) and consumption over biomass (Q/B) ratios per trophic group for each study site. Conversion factors came from Brey [55] and Opitz [89]. FFS is French Frigate Shoals.
(TIF)

Table S2 Fish species included in the Ecopath models per each functional group. Inclusion was based on presence data from daytime visual surveys in shallow water (<30 m) hard-bottom habitats (CRED unpubl. data).
(TIF)

Table S3 Ecopath input data and resulting parameters for the 33 functional groups for French Frigate Shoals. Values calculated by EwE are shown in bold.
(TIF)

Table S4 Ecopath input data and resulting parameters for the 33 functional groups for the Kona Coast. Values calculated by EwE are shown in bold.
(TIF)

Table S5 Ecopath input data and resulting parameters for the 33 functional groups for O’ahu. Values calculated by EwE are shown in bold.
(TIF)

Table S6 Diet composition matrix of the functional groups included in a reef system around French Frigate Shoals. Import indicates feeding outside of the modeled area. Numbers in column headings (predators) correspond with numbers in row headings (prey), e.g., group 19 represents corals. The sum of the diet composition (column) equals to 1. CCA is crustose coralline algae. Column headings correspond to the row headings.
(TIF)

Table S7 Diet composition matrix of the functional groups included in a reef system along the Kona Coast of Hawai’i. Import indicates feeding outside of the modeled area. Numbers in column headings (predators) correspond with row numbers (prey). The sum of the diet composition (column) equals to 1. CCA is crustose coralline algae. CCA is crustose coralline algae. Column headings correspond to the row headings.
(TIF)

Table S8 Diet composition matrix of the functional groups included in a reef system around O’ahu. Import indicates feeding outside of the modeled area. Numbers in column headings (predators) correspond with row numbers (prey). The sum of the diet composition (column) equals to 1. CCA is crustose coralline algae. CCA is crustose coralline algae. Column headings correspond to the row headings.
(TIF)

Text S1 Supplementary material specific to the 33 functional groups. Explanation and sources of used Ecopath values in the three Hawaiian studies and diet composition of each model.
(DOCX)
