# Supplementary figures and images for: Comparison of Coral Reef Ecosystems along a Fishing Pressure Gradient
Source: PLoS One. 2013 May 30;8(5):e63797. doi: 10.1371/journal.pone.0063797 (PMC3667803; doi:10.1371/journal.pone.0063797)

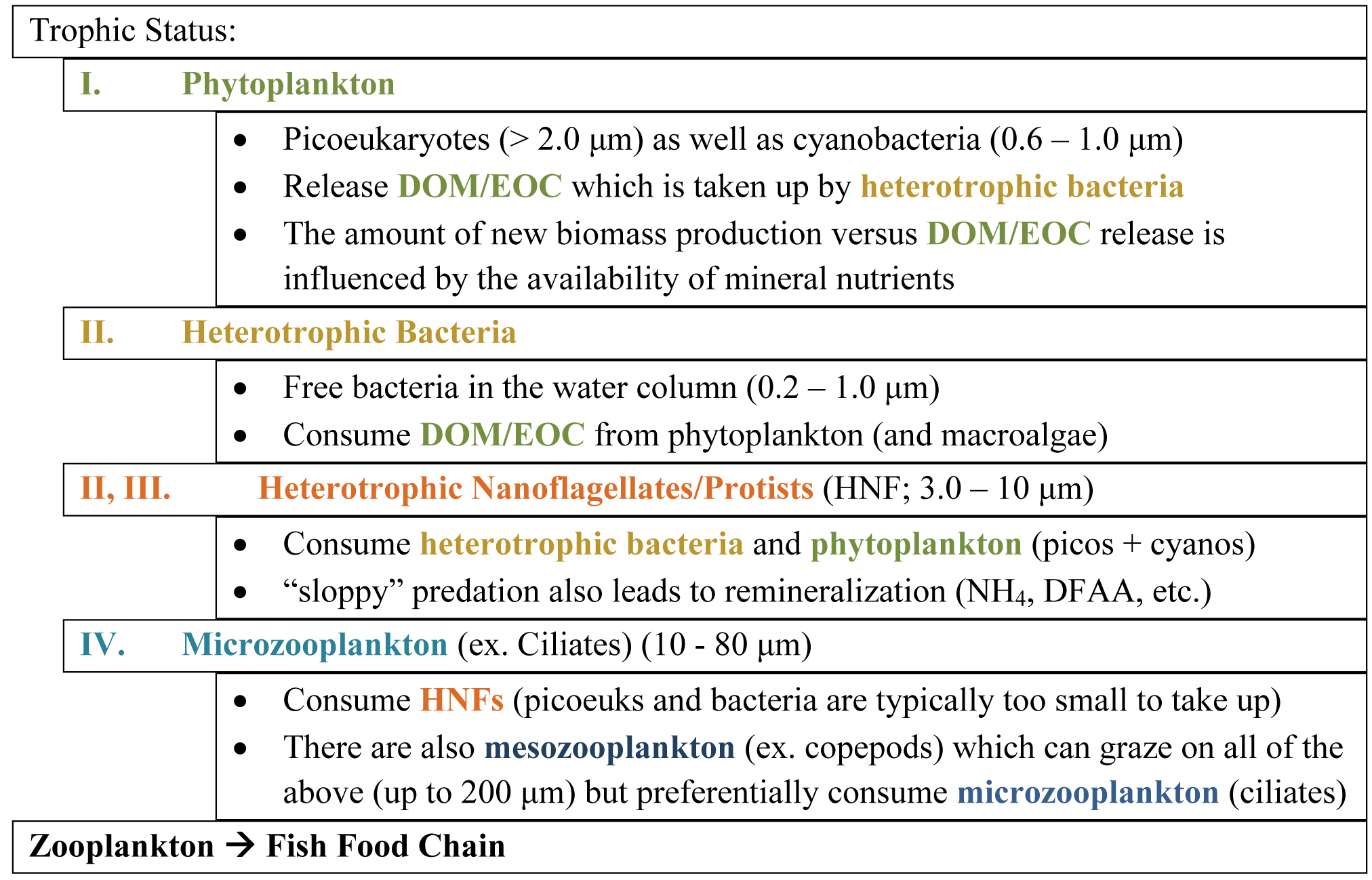

Supplement: Figure S1 — Schematic representation of the marine microbial loop. DOM is dissolved organic matter; EOC is extracellular organic carbon; DFAA is dissolved free amino acids; HNF is heterotrophic nanoflagellates or protists. Diagram created by Tracy McDole, San Diego State University. (TIF) [file pone.0063797.s001.tif]

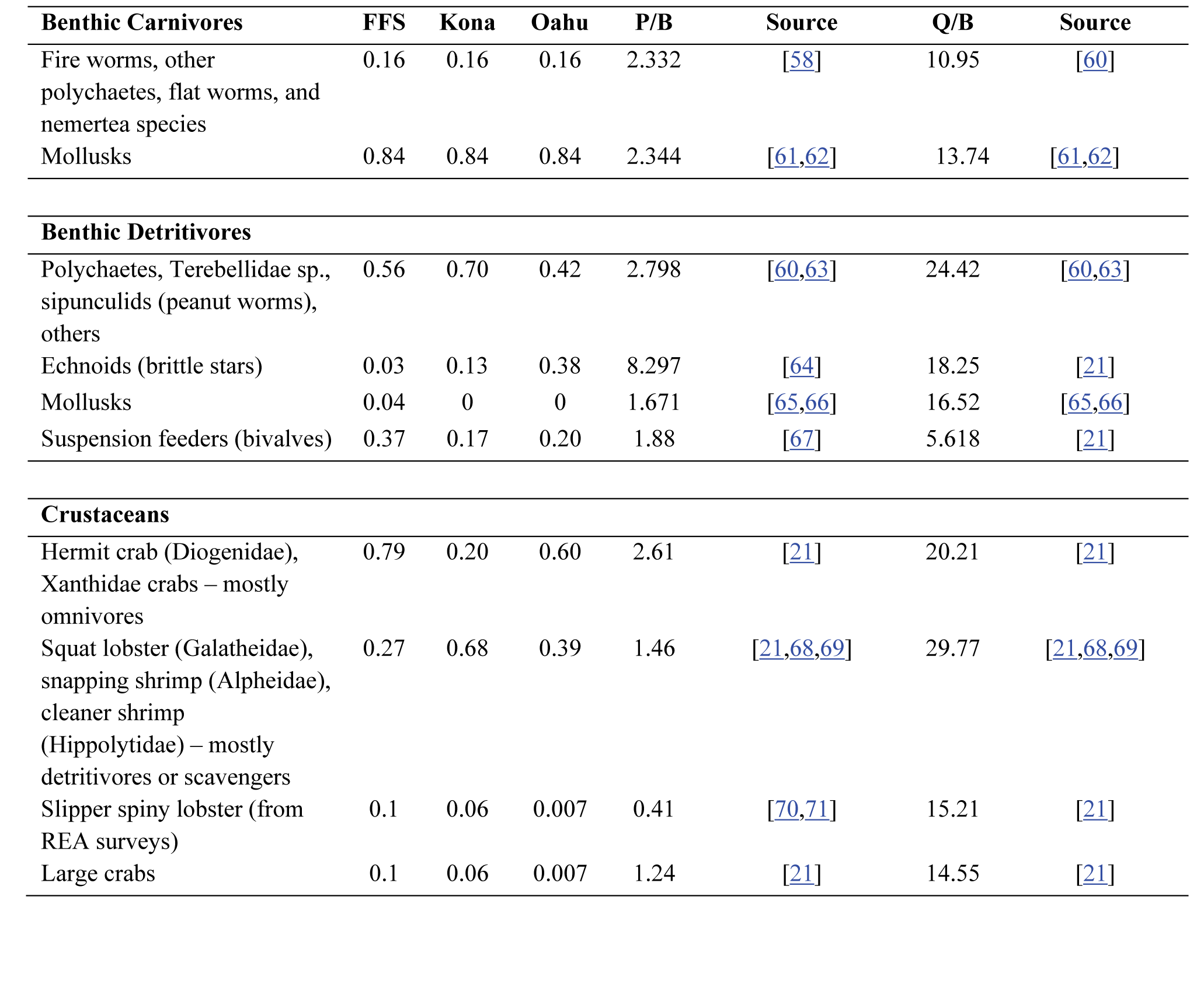

Supplement: Table S1 — The weighting factors in percentage and input values with their source for the production over biomass (P/B) and consumption over biomass (Q/B) ratios per trophic group for each study site. Conversion factors came from Brey [55] and Opitz [89]. FFS is French Frigate Shoals. (TIF) [file pone.0063797.s002.tif]

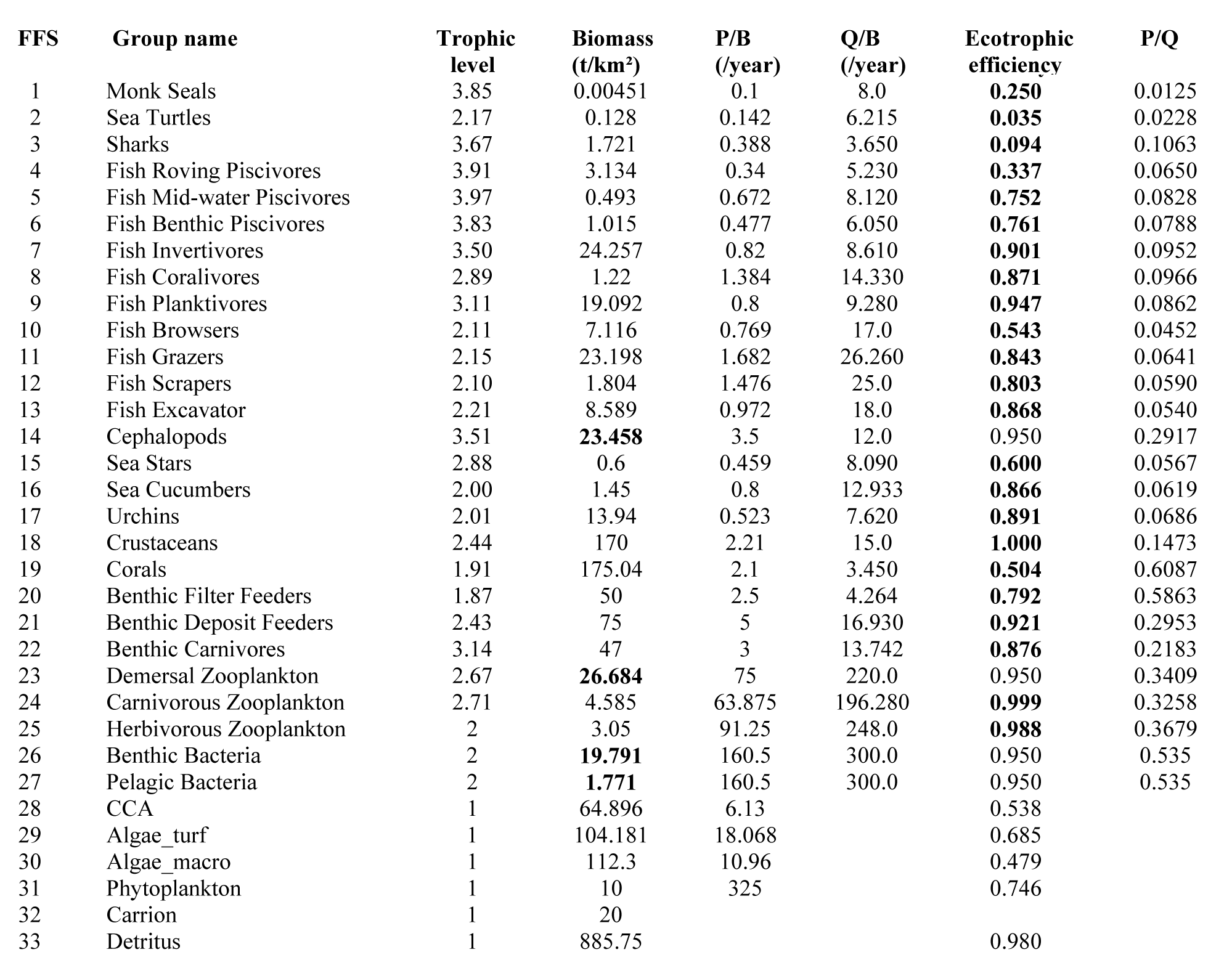

Supplement: Table S3 — Ecopath input data and resulting parameters for the 33 functional groups for French Frigate Shoals. Values calculated by EwE are shown in bold. (TIF) [file pone.0063797.s004.tif]

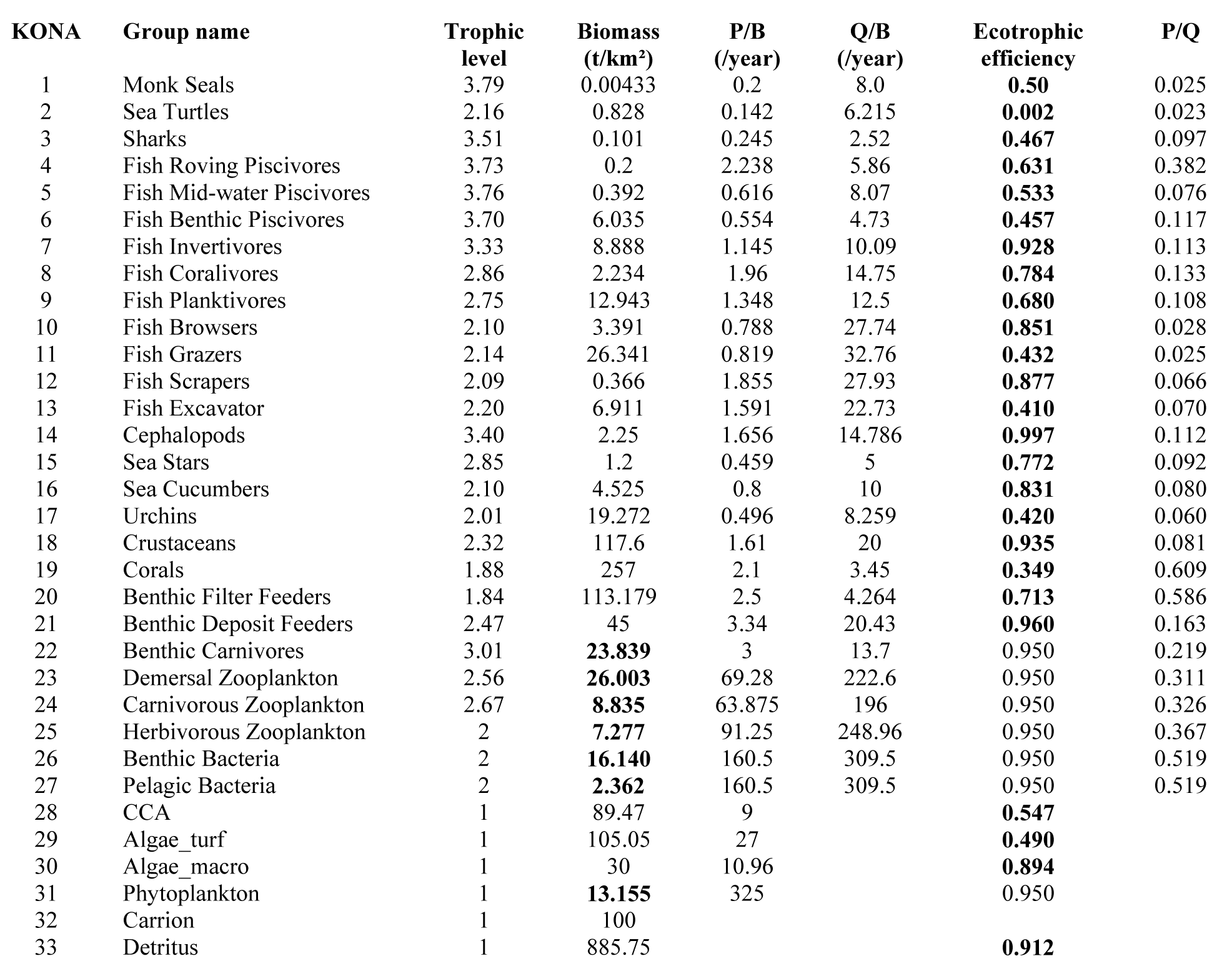

Supplement: Table S4 — Ecopath input data and resulting parameters for the 33 functional groups for the Kona Coast. Values calculated by EwE are shown in bold. (TIF) [file pone.0063797.s005.tif]

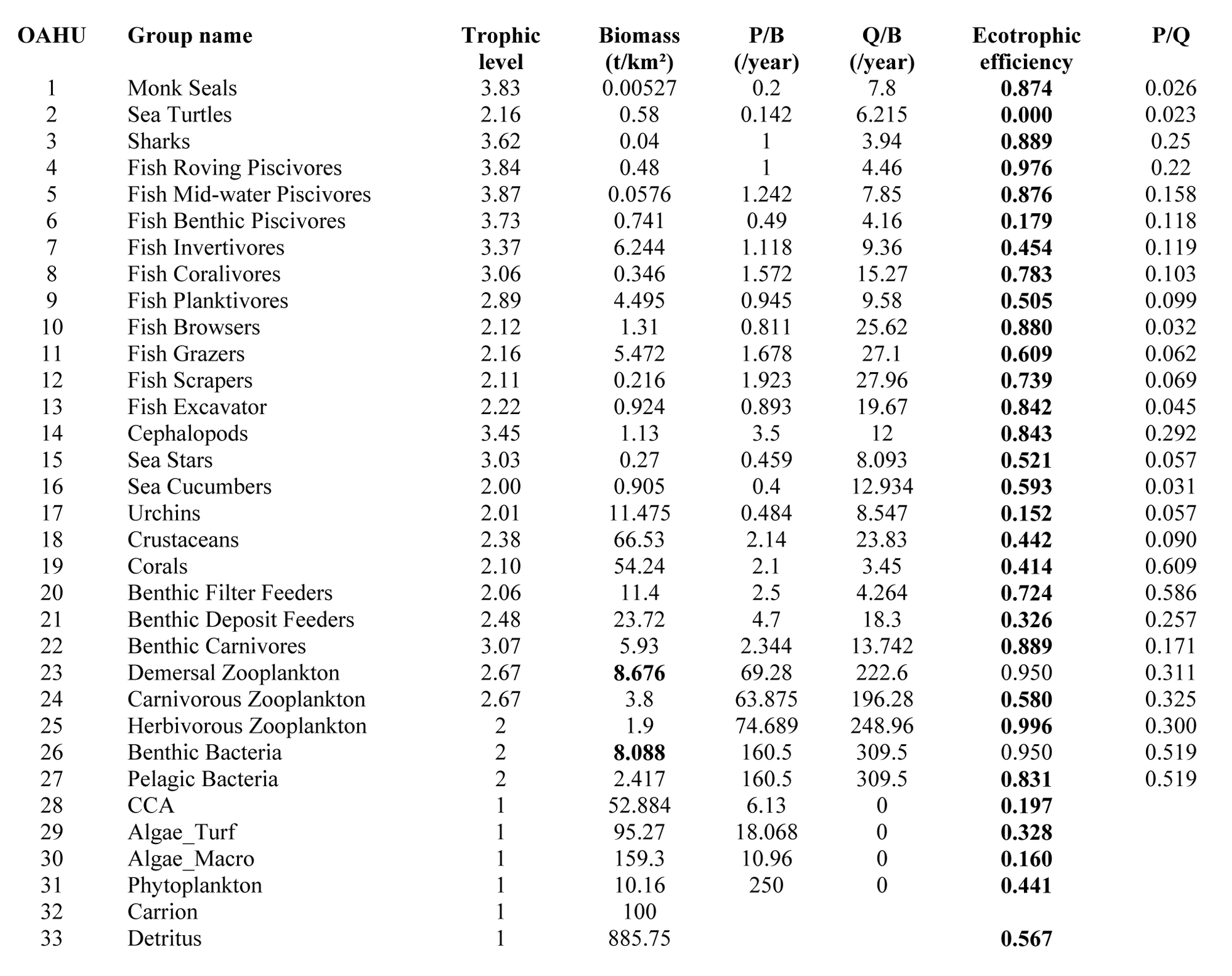

Supplement: Table S5 — Ecopath input data and resulting parameters for the 33 functional groups for O’ahu. Values calculated by EwE are shown in bold. (TIF) [file pone.0063797.s006.tif]

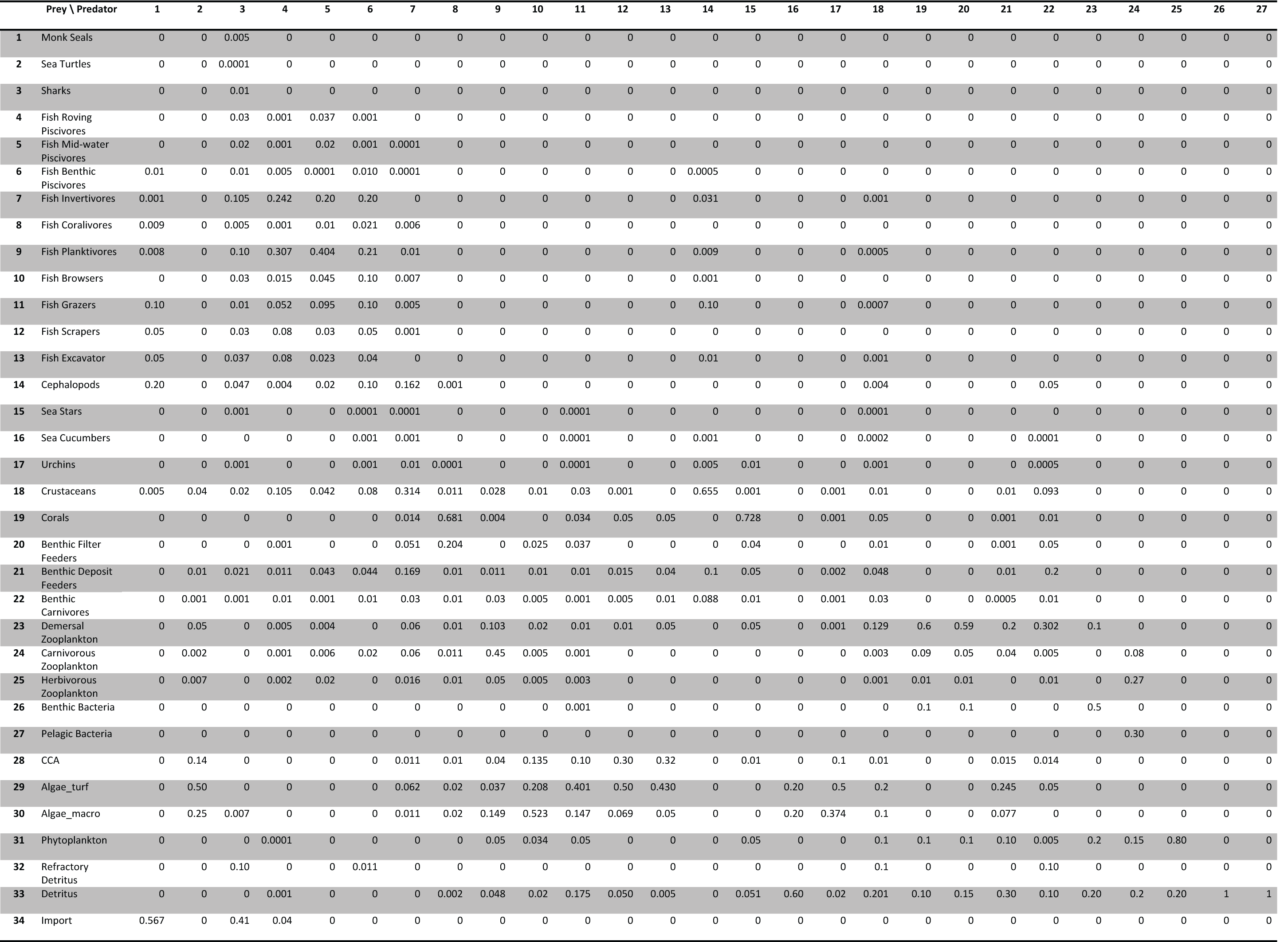

Supplement: Table S6 — Diet composition matrix of the functional groups included in a reef system around French Frigate Shoals. Import indicates feeding outside of the modeled area. Numbers in column headings (predators) correspond with numbers in row headings (prey), e.g., group 19 represents corals. The sum of the diet composition (column) equals to 1. CCA is crustose coralline algae. Column headings correspond to the row headings. (TIF) [file pone.0063797.s007.tif]

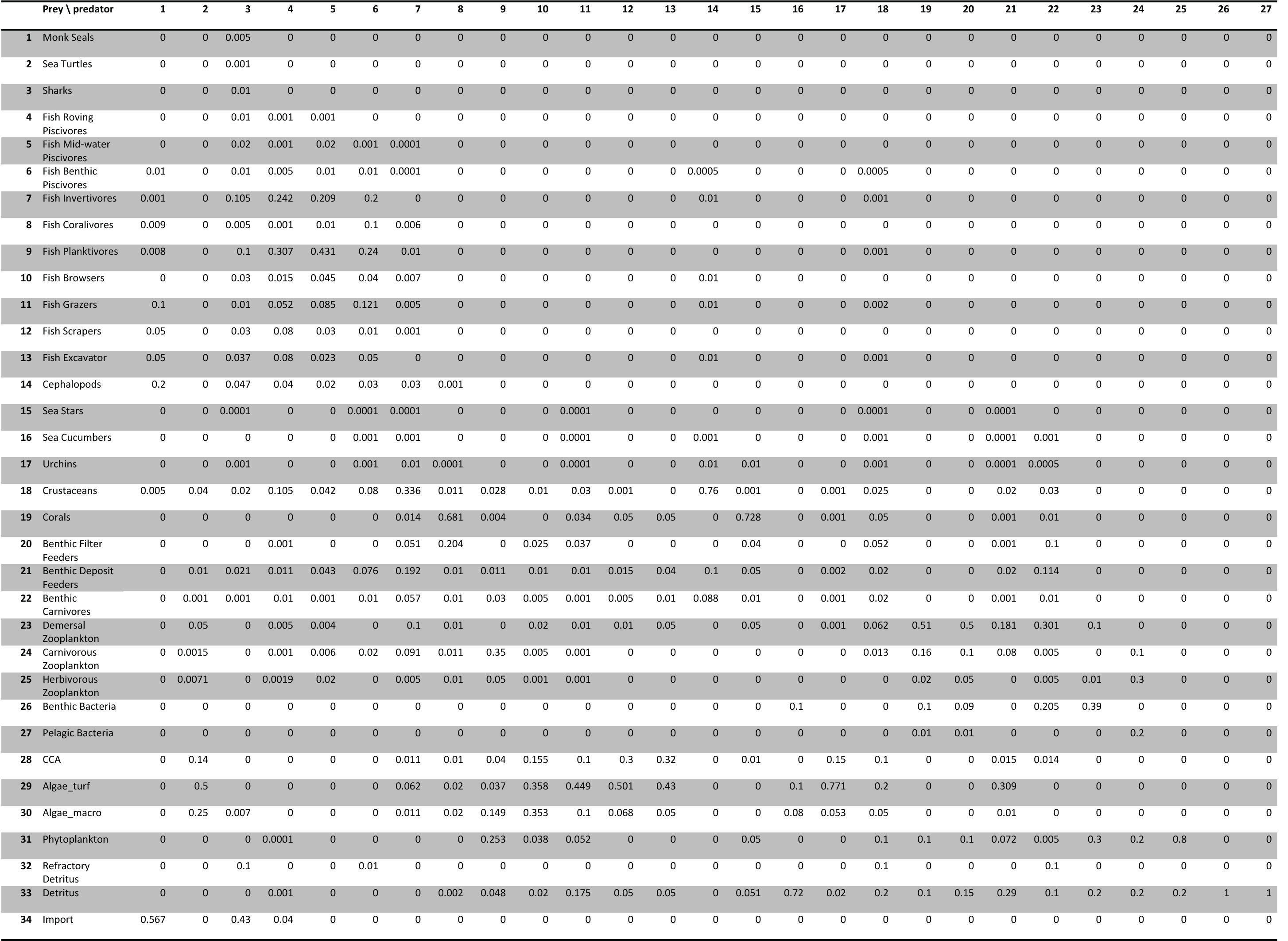

Supplement: Table S7 — Diet composition matrix of the functional groups included in a reef system along the Kona Coast of Hawai’i. Import indicates feeding outside of the modeled area. Numbers in column headings (predators) correspond with row numbers (prey). The sum of the diet composition (column) equals to 1. CCA is crustose coralline algae. CCA is crustose coralline algae. Column headings correspond to the row headings. (TIF) [file pone.0063797.s008.tif]

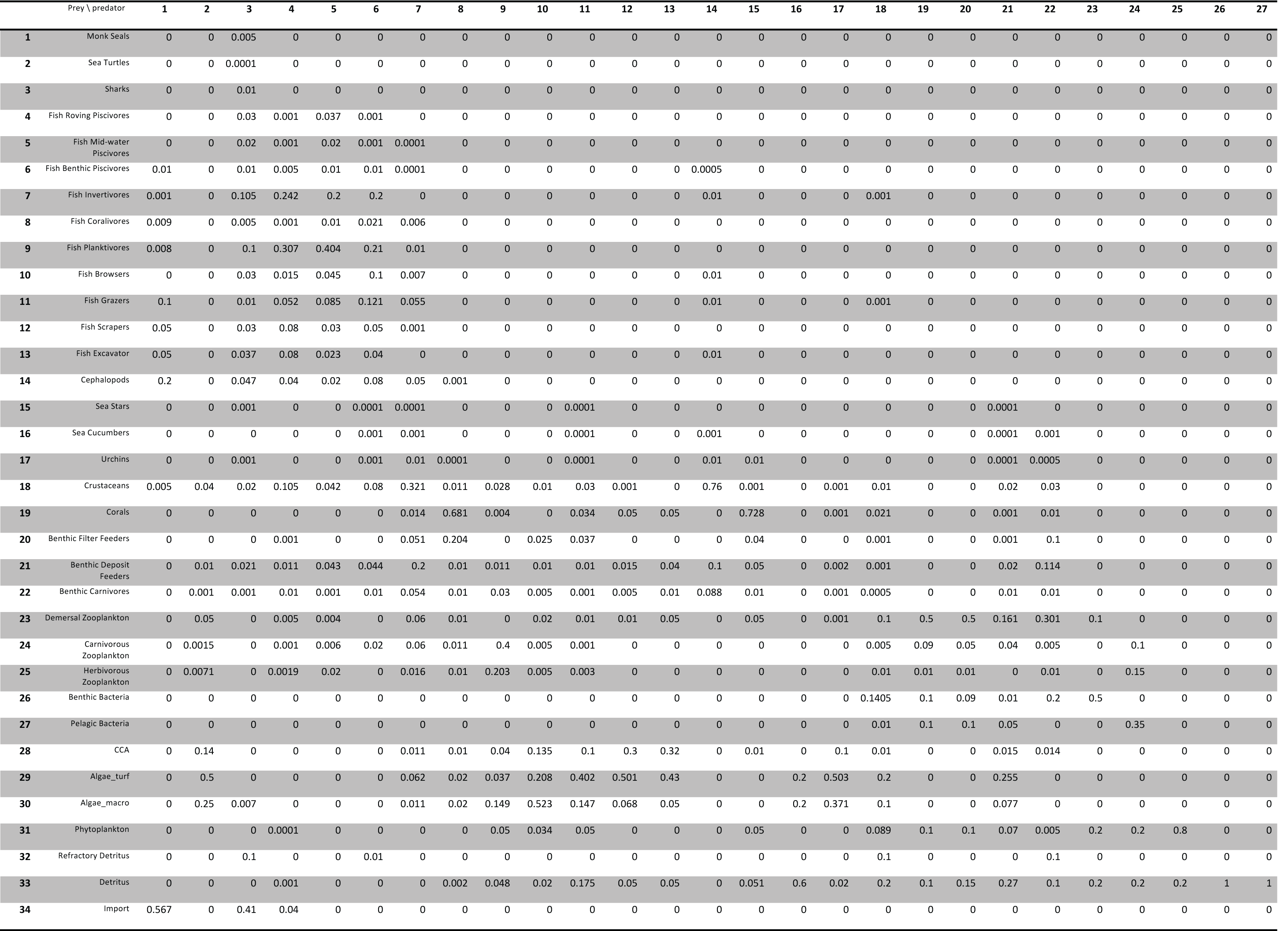

Supplement: Table S8 — Diet composition matrix of the functional groups included in a reef system around O’ahu. Import indicates feeding outside of the modeled area. Numbers in column headings (predators) correspond with row numbers (prey). The sum of the diet composition (column) equals to 1. CCA is crustose coralline algae. CCA is crustose coralline algae. Column headings correspond to the row headings. (TIF) [file pone.0063797.s009.tif]
