# Supplementary material for: Comparison of Coral Reef Ecosystems along a Fishing Pressure Gradient
Source: PLoS One. 2013 May 30;8(5):e63797. doi: 10.1371/journal.pone.0063797 (PMC3667803; doi:10.1371/journal.pone.0063797)
Supplement: Text S1 — Supplementary material specific to the 33 functional groups. Explanation and sources of used Ecopath values in the three Hawaiian studies and diet composition of each model. (DOCX) [file pone.0063797.s010.docx]

**Supporting Information**

**Comparison of coral reef ecosystems along a fishing pressure gradient**

**Mariska Weijerman, Elizabeth Fulton, Frank Parrish**

This supplement provides detailed information with regards to the species composition of the 33 functional groups and the input and output parameters of the Ecopath models for the coral reef systems around French Frigate Shoals (FFS), Northwestern Hawaiian Islands, along the Kona Coast of Hawai`i Island, and around O`ahu.

**S1. SUPPLEMENTARY MATERIAL** specific to the 33 functional groups

S1.1 Detritus

In the models, detritus represents the pool of dead organic material, including particulate and dissolved organic matter. Detritus standing stock was estimated as a function of primary production (PP in gC/m^2^/y ) and euphotic depth (EE in m) using an empirical equation [1]:

logD=-2.41+0.954*logPP+ 0.863*logE

The euphotic depth was estimated to be 110 m [2]. Primary production estimates for Hawaiian coral reefs range from 3895 g/m^2^ [3], to 4513 g/m^2^ [4], and 6100 g/m^2^ [5]. We used the mean of these three estimates to estimate the detritus standing stocks.

S1.2 Carrion

We also included carrion and estimated it to be higher in the populated islands (100 t/km^2^), as fishermen discard unwanted parts of their catch, compared to the unpopulated FFS (20 t/km^2^).

S1.3, 1.4 Pelagic and Benthic Bacteria

We tried to include the microbial loop (Fig. S1) but were constrained by the available data.

Biomass for pelagic bacteria came from water samples collected at the surface and about 1 m above the reef by the Pacific Reef Assessment and Monitoring Program (RAMP; CRED unpubl. data). For benthic bacteria we let Ecopath calculate the biomass. The P/B estimate for both pelagic and benthic bacteria was the mean reported by Gribble [6] and Tsehaye and Nagelkerke [7]; the Q/B estimate for both bacteria groups was the mean of Hatcher [8], Gribble [6], Opitz [9] and Tsehaye & Nagelkerke [7].

S1.5 Phytoplankton

Water samples were collected at the surface and about 1 m above the reef by the Pacific RAMP (CRED unpubl data) from 2005 to 2010; *n* = 6-13 per year around Hawai`i, 3-6 per year around O`ahu and 3-6 per year around FFS. Biomass estimate of phytoplankton in the water column was derived from chlorophyll-*a* concentration in the water samples using a conversion factor of 0.3 Chl-*a* per 100 phytoplankton biomass and the mean reef depth derived by the Pacific RAMP to calculate the wet weight in t/km^2^.

The P/B ratio (115/y) was the mean from O`ahu [10] and an offshore research station off the coast of north O`ahu [11,12]. However, this estimate proved to be too low to sustain productivity at higher trophic levels in the models so we increased it to 325/y reported by Wabnitz et al. [3] for the Kona Coast of Hawai`i.

**S1.6-1.8 Zooplankton**

As the annual phytoplankton production cycle shows little variation in the tropical seas, zooplankton biomass follows these cycles and is more or less stable throughout the year unlike the temperate oceans where there is a summer bloom following the spring bloom of phytoplankton. For the Hawai`i Ecopath models, we combine holoplankton, meroplankton and gelatinous zooplankton in carnivorous and herbivorous zooplankton and show separate estimates for demersal (or benthic) zooplankton. Zooplankton composition differs for the major zooplankton groups with depth. In both oceanic and demersal zooplankton, copepods make up the highest contribution in the total zooplankton abundance (93%-96%) but in biomass this is 30%-36% [13]. Decapods (27.8%), polychaetes (26.2%) and chaetognaths (9.8%) make up the next 65% in biomass. Assuming all copepods are grazers and the rest are all carnivorous, we used a composition of carnivorous: herbivorous zooplankton of 30:70. Diet data came from various published sources [14-18].

S1.6 Herbivorous Zooplankton

Biomass for zooplankton was based on Roman et al. [11] who estimated the mesozooplankton biomass from Station HOT located north of O`ahu at 23.84 mmolC/m^2^ which corresponds to 6.36 g/m^2^ using a conversion factor of 0.45 from carbon to dry weight [19] and a factor 10 from dry weight to wet weight for organic tissue [9]. Copepods represent approximately 30% of the total zooplankton biomass. Herbivorous biomass, therefore, was estimated at 1.910 g/m^2^.

The Q/B value (248/y) was the average of the mean of two estimates: (1) the equally weighted mean of coastal and open ocean, derived from Calbet et al. [19] for small copepods (383/y), and (2) the estimate by Roman et al. [11] for copepods of 115 per year. The two estimates from Calbet et al. [19] were based on (i) consumption in the mesotrophic Kaneohe Bay of 100%-260% of their body weight daily; and (ii) in the oligotrophic open ocean, which has an order of magnitude lower biomass of phytoplankton and, hence, less food for the grazing copepods (so they corrected the daily consumption to 3% body weight but stated that this value seemed unrealistically low so we used 30% which was found in the same study under food limitation). Le Borgne [20] showed an estimate of 265.813 per year for the Coral Sea which is comparable to our value. The P/B value for herbivorous zooplankton (91.25 per year) is based on Le Borgne [20].

S1.7 Carnivorous Zooplankton

Biomass for carnivorous zooplankton was based on Roman et al. [11] who estimated total mesozooplankton biomass at 23.84 g/m^2^ from Station HOT located north of O`ahu of which 60% (3.810 g/m^2^) represents carnivorous zooplankton [13].

The P/B value for carnivorous zooplankton 63.88/y is based on Le Borgne’s [20] review of P/B estimates for carnivorous zooplankton from the Coral Sea, which has similar temperatures as Hawai`i, that ranged from 15% to 20% daily; using the mid value of this range, we estimated the P/B ratio to be 29.2/y. The Q/B ratio of 196.28/y was based on Ainsworth et al. [21].

S1.8 Demersal Zooplankton

Demersal zooplankton biomass was estimated at 4.5 mg C/m^3^ in Discovery Bay, Jamaica [17] and 8.1-21.4 mg C/m^3^ in Conch Reef, Florida [13]. This higher estimate in Florida was based on the greater amount of large zooplankton species in that area [13]. We allowed Ecopath to calculate our biomass with an EE of 0.95.

The Q/B and P/B ratios were the means of the carnivorous and herbivorous zooplankton groups.

S1.9 Turf Algae

Biomass estimate for turf algae was 13.5 gC/m^2^ based on Smith et al. [22] for a reef system off Puako, Hawai`i, which was corrected for turf algal cover at each study site and converted to wet weight [9]. Another estimate came from field studies off Waikiki Beach, O`ahu (T. Sauvage, pers. comm.). They estimated between 205 and 370 gWW/m^2^ of turf biomass and this estimate was corrected for turf algal cover at each site derived from Pacific RAMP and Hawai’i State Division of Aquatic Resources (DAR) data. For the models we used an average of these two values.

The P/B ratio (18.068/y) was based on estimated daily turnover time for turf algae by Klumpp and Klumpp and McKinnon [23]. Miller et al. [24] estimated a productivity of 182.5 gC/m^2^/y for turf algae on a temperate reef in California and a biomass of 11 gC/m^2^ yielding a P/B ratio of 16.591/y. Wabnitz et al. [3] reported a P/B ratio of 19/y for a reef system on the Kona Coast of Hawai`i. Carpenter et al. [25] estimated a productivity of 10 ugO_2_/μg Chl-*a*/h which corresponds to a P/B ratio of 18.25/y. All estimates compare well with our estimate.

S1.10 Calcareous Coralline Algae (CCA)

Biomass estimate for CCA was 78 gDW/m^2^ based on Smith et al. [22] for a reef system off Puako, Hawai`i, which was corrected for CCA cover at each study site and converted to wet weight [9]. Littler and Murray [26] estimated primary production for coralline algae of 0.5–2.6 gC/m^2^/d for a Hawaiian fringing reef (Waikiki, O`ahu); Hatcher’s [27] estimate was comparable with 1.8 gC/m/d, as was Marsh’s [28] estimate of 0.65-2.15 gC/m^2^/d, and Anthony et al.’s [29] estimate of 2.1 gC/m^2^/d. Using an average daily productivity of 1.725 gC/m^2^/d and a biomass value based on Odum and Odum [30] corrected for 6.42% CCA cover for Hawaiian reefs (Pacific RAMP, DAR data) gave us a P/B ratio of 6.130/y. This ratio is comparable with the turnover time estimated by Klumpp and McKinnon [23] of 6.753/y and the P/B ratio calculated by Hatcher [31] of 6.277/y but higher than the ratio given by Wabnitz et al. [3] of 1.77.

S1.11 Macroalgae (MA)

Biomass estimate was based on the wet weight (WW) of high turf from Smith et al. [22] (600 gWW/m^2^) and this value was increased by 150% to get a more accurate estimate of the taller macroalgae and subsequently corrected for macroalgal cover at each study site (Pacific RAMP and DAR data).

Macroalgal productivity was estimated at 1.44 gC/m^2^/d by Smith et al. [22] for a coral reef system in Puako, Hawai`i. Using a conversion factor from Opitz [9] and a biomass value based on Odum and Odum [30] yielded us a P/B ratio of 11.963/y for fleshy macroalgae. For calcareous macroalgae, we used the productivity estimate from Payri et al. [32] for three *Halimeda* species on a reef in Moorea Island, Tahiti, which yielded a P/B ratio of 9.96/y and averaged the two values (10.96/y). P/B values were comparable to 10.5/y from Ainsworth et al [21]; 9.8 per year from Wabnitz et al. [3], and 12.8 per year from Carpenter et al. [25].

S1.12 Benthic Filter Feeders

In oligotrophic waters, reefs are dominated by animal-algal symbioses whereas these symbioses are gradually replaced by heterotrophic animals especially filter feeding sponges, ascidians and mollusks as nutrient levels increase [33,34]. For example, in the oligotroph oceanic waters of the Great Barrier Reef (GBR), 70% of the sponges are phototrophic [35]. Large sponges, common on Caribbean and Indo-Pacific reefs are absent from Hawaiian reefs; however, small encrusting sponges are abundant and because of their high pumping rate (up to 70% efficiency in phytoplankton clearance; [36,37]), they are probably the main filter feeders in Hawai`i. The most dominant soft corals are *Sarcothelia edmonsoni*, found in waters between 0 and 5 m. Anemones are also small in size and often cryptic in Hawai`i. Zooanthids are another sessile filter feeder present in Hawaiian waters and included in our functional group of benthic filter feeders. We assumed that 50% of the productivity contributed to the primary productivity. Cover data for benthic filter feeders was derived from Pacific RAMP and DAR data.

For sponge consumption, we used consumption rates of sponges on oligotrophic reefs in the GBR [35]. Consumption was estimated at 0.084 gC/m^2^/d for a sponge biomass of 76.2 g/m^2^. With a conversion factor of C to WW = 10% and subtracting 22.5% inorganic sponge biomass [30], the Q/B ratio was 4.024/y. This value was comparable to the total Q/B value for an inner, outer, and oceanic reef system in GBR of 4.701/y [35]. The P/B value was based on the same study, yielding a ratio of 0.894/y.

Fabricius and Domisse [38] calculated a consumption rate of the soft coral *Sinularia* sp. from the GBR at 2.5 gC/m^2^/d for a high standing stock biomass of 200 g AFDW/m^2^ (where AFDW stands for ash-free dry weight). Using a conversion factor of 10% for organic tissue to WW of zooplankters and 8% for AFDW to WW (from Brey for bryozoas)[58] and subsequently subtracting 22.5% of inorganic weight [30], the Q/B was calculated at 4.710/y. For octocoralia, the P/B ratio of 0.213/y was based on the natural mortality of two gorgonian species from the Caribbean [39].

We calculated the weighted means for Q/B and P/B for the functional group of benthic filter feeders. This resulted in a Q/B ratio of 4.26/ y which is lower than the 12/y given by Gribble [6] for a reef system in the GBR but comparable to the 4.63 per year for octocorals from the Kona Coast of Hawai`i [3], and a P/B of 0.76/ y which is comparable to Gribble’s [6] estimate of 0.8/y but higher than Wabnitz et al.’s [3] estimate (0.2/y).

S1.13 Corals

There are 69 species of corals identified in Hawai`i [40] with taxonomic uncertainty of some species [41]. Of these identified species only a handful dominate the benthos and are important reef-building species: *Pocillopora* spp., *Montipora* spp., *Porites lobata*, and *P. compressa*. In the model, we represented corals as coral holobionts being facultative producer/hetrotroph; the holobiont is the collective community of coral host and its metazoan, protest, and microbial symbionts.

Based on Odum and Odum [30] biomass of heterotrophic tissue (polyps) and zooxanthellae was 0.021 and 0.058 gm DW/cm^2^ (average of massive and branching corals). Using a conversion factor of 22.5% for polyps and 17.5% for zooxanthellae for dry weight to wet weight gave us a biomass estimate of 158 g/m^2^ for 100% coral cover and this value was corrected for coral cover at each study site (Pacific RAMP and DAR data). We further assumed that corals contribute 50% to primary productivity [42]. Besides their phototrophic dependence, corals are also heterotrophs and have been shown to feed on a range of food types, e.g., zooplankton, microzooplankton, bacteria, sediment and suspended particulate matter, the latter comprising components from all particle types [42,43].

Heidelberg et al. [17] showed that zooplankton biomass was high throughout the night, not only at dawn and dusk, on a forereef of Jamaica. Palardy et al. [43] showed that zooplankton biomass was patchy over a reef and dependent on the lunar cycle. We assumed an 8-hour feeding period for the calculation of consumption. We further assumed that the average size of the ingested zooplankton was 0.3 mm [42] and used an empirical formula for ‘other –than copedpod - zooplankton” to convert size (in mm) to biomass in μg C [17]:

LN(biomass) =1.46*LN(Size)+1.03

and an organic C to WW conversion factor of 10 [9] which gave us a WW of 4.8 μg for one zooplankton. On average, *Porites lobata* polyps eat 28, *P. compressa* eat 16, and *Montipora capitata* eat 8 zooplankters/g AFDW polyp/h [42]. From Pacific RAMP surveys we obtained the coral species composition of *P. lobata* 8.6%, *P compressa* 6.4%, and *Montipora* sp. 4.3% for the main Hawaiian Islands. Using this composition, we calculated the weighted mean zooplankton feeding rate as 19.6 zooplankters/g AFDW polyp/h. This corresponds to a daily Q/B ratio of 3.45/y which we used for all three systems.

This value is comparable with the 3.6/y from Ainsworth et al. [21] and a little higher than 2.1 per year from Wabnitz et al. [3]. We further assumed that the production/consumption ratio was 0.6 [21] and calculated the P/B ratio (2.1 per year).

**S1.14-1.20 Invertebrates**

Biomass data for cryptic and benthic invertebrates was difficult to obtain. For the benthic epifauna we used the data from the Pacific RAMP, but these are all daytime surveys and many invertebrates hide during the day so it is likely an underestimation. For the cryptic species, we used the community composition from nine 1-year deployments of Autonomous Reef Monitoring Structures (ARMS; www.pifsc.noaa.gov/cred/arms.php. Accessed 2010 Dec) off south O`ahu. As these deployments were only from one location and sessile invertebrates were not analyzed yet, we do not have a complete picture of the invertebrate community but it was the best available data.

When grouping all animals retrieved from ARMS in carnivores and others (detritivores, scavengers, filter feeders, omnivores), the community composition was 16.9%:83.1% = ~ 1:5. Mean number of invertebrates was 373 distributed over the 10 plates of 1 ARMS unit. The area of 1 plate is 0.052 m^2^, assuming animals can attach/inhabit the top and bottom of the plates, total area is about 1 m^2^. Crustaceans ranged in size between a few millimeters for amphipods to 2.5 cm for snapping shrimp with the most abundant group being the hermit crabs; polychaetes could reach as much as 10 cm for the abundant spaghetti worms; gastropods ranged in size from 0.5 cm to 2 cm with the most abundant family, the carnivorous muricoidae. Assuming a mean weight of 0.5 g per animal gives us a total biomass for infauna and epifauna of 186.5 g/m^2^ which is similar to the estimate of Smith et al. [22]. Smith et al. [22] determined the number of mobile epifaunal invertebrates on a Hawaiian coral reef system on the Kona Coast of Hawai`i and estimated the total number of crustaceans to be 37.7 (SE 3.2), polychaetes 7.4 (SE 1.1) and gastropods 9.4 (SE 1.1) ind/50 cm^2^ and all were a few millimeters in length (J. Smith, pers.comm., 31 Jan 2012). These values correspond to a wet weight of 113.1, 22.2 and 28.2 g/m^2^ respectively, assuming an average individual weighs 15 mg, and yields a total weight of 163.5 g/m^2^. Kroeker et al. [44] estimated the benthic invertebrate community from the Mediterranean Sea to be approximately 95 g/m^2^.

Species were grouped in the various functional groups according to their diet [16,37,45-54]. Final groups were checked by invertebrate experts (G. Paulay, University of Florida; S. Eberhardt, University of Hawai`i; M. Timmers, JIMAR) and compared with group compositions from Enochs [55] and Enochs et al. [56].

S1.14-1.16 Benthic Carnivores, Detritivores and Crustaceans

Using the community composition from ARMS and the total biomass value by Smith et al. [22], we calculated a crustacean biomass of 113.1 g/m^2^, carnivorous invertebrate biomass of 10.08 g/m^2^ and a detritivorous invertebrate biomass of 40.32 g/m^2^.

P/B and Q/B ratios came from literature or empirical relationships with input parameters from literature (Table S1). The P/B depends highly on the mean annual temperature, mean individual weight, and swimming capacity of marine macroinvertebrates [57]; therefore, if we did not find any reported values in the literature, we used an empirical model established by Brey [58] that includes those aspects:

Log (P/B) = 7.947-2.294 * log(M) – 2409.856 * 1/(T+273) + 0.168 * 1/D + 0.194 + 0.180 * Infauna/Epifauna + factor * taxon - 0.062 + 582.851 * log(M) * 1/(T+273)

M is the mean individual body mass in kilojoules, D is the water depth in meter, infauna equals 1, epifauna equals 0, motility and taxon are also taken into account with taxon being Annelida, Crustacea, Echinodermata or Insecta.

For the Q/B ratio, we used the empirical relationship established by Cammen [59]:

C = 0.381*W^0.742

C is the daily consumption in mg dry weight (DW), W is the mean body weight in mg DW.

Based on the species or family composition of the benthic small invertebrates derived from ARMS and visual surveys at shallow-water (< 30 m) forereef sites, we obtained a weighting factor for the calculation of P/B and Q/B per functional group of invertebrates (Table S1).

S1.17 Sea Cucumbers

Two orders of sea cucumbers, class Holothuroidae, are common on Hawaiian reefs, Aspidochirotida including family Holothuriidae and Stichopodidae, and Apodida, family Synaptidae [51]. Most sea cucumbers are nocturnal and detritivores. They range in size between about 4 inches (*Holothuria* *padalis*) and about 3 ft (*Opheodesoma spectabilis*) and the majority of the Hawaiian species range between 1 and 2 ft [51]. Larger organisms are easily visible clinging to rocky reefs or on sand. Small individuals hide beneath stones, in crevices or partially buried in sand. Ebert [72] estimated a mean wet weight between 25 and 156 g for an average 5-year-old *Holothuria altra* with a maximum length of 32 cm and a maximum age of 9 years weighing 1352 g. We used the mean values for *Holothuria* *altra* [72] to calculate P/B and Q/B ratios for our sea cucumber functional group, yielding a value of 0.240/y based on Brey [58] and 12.934/y based on Cammen [59]. The P/B ratio is lower than other reported estimates. Recalculating the P/B based on maximum age and weight from Ebert [72] gave us 0.393/y. As the P/Q ratio was just 3% but should be between 5% and 30% [73], we doubled the P/B, which made it also more comparable to the value reported by Ainsworth et al. [21] and this increased the P/Q to 6%.

Diet data came from Uthicke et al. [53].

S1.18 Sea Stars

Biomass and abundance data for three sea star species came from Pacific RAMP towed-diver surveys and DAR belt-transect surveys conducted from 2005 to 2010 (CRED and DAR unpubl. data). The most frequently observed species were *Linckia multiflora,* *Mithrodia fisheri*, *Acanthaster* *planci* (crown-of-thorns seastar, COTS), *Linckia guildingi*, and *Culcita novaeguineae* with *Linckia* sp. being most abundant in Kona and FFS and COTS around O`ahu. Diameter was converted to weight using the equation in Birkeland and Lucas [74]:

WW(g) starfish = 0.1609 * Radius (cm)^2.893;

However, for *Culcita* sp., the radius to weight relationship of Yamaguchi [75] was used. Weighted biomass based on mean weight (assuming 2/3 of maximum radius) and overall density was calculated for each study area.

For COTS, the max age was 8 y [76] and max weight estimated using the max diameter (45 cm) from Hoover [51] gave us a P/B ratio of 0.442/y using Brey’s empirical relationship [58]. The P/B values for *Linckia* sp. and *Culcita novaegineae* were also computed using the same max age as for COTS and a max radius of 14 cm for *Culcita novaeguinea* and 15 cm for *Linckia* sp. [51]. Using these values and Brey’s [58] conversion factors (WW-AFDW = 0.124; J/mgAFDW = 20.81), the P/B ratio was calculated at 0.461/y and 0.463/y. For each study site a weighted mean was calculated based on the sea star species composition derived from the visual surveys.

Q/B ratio for *Culcita* and *Linckia* was based on the coral consumption rate of 28 cm^2^/d estimated for *Culcita* [77] using the biomass of coral polyps from Odum and Odum [30] of 0.021 gDW/cm^2^ and a conversion factor of DW to WW of 22.5%. This yielded a Q/B ratio for *Culcita* of a 9.127/y and 7.476/y for *Linckia*. Consumption was estimated at 5.3 m^2^/y for COTS [78] resulting in a Q/B ratio of 11.997/y. The weighted mean was estimated per study site.

Diet data came from Uthicke et al [53] and Bell [79].

S1.19 Cephalopods (CEP)

From Pacific RAMP surveys conducted from 2002 to 2010 (CRED unpubl. data) we estimated a mean abundance of octopus per study site. We assumed the mean weight to be 750 g to calculate the biomass per site.

Ingestion rate varied between 2% and 6% body weight per day [80]. We took the average and calculated Q/B at 14.6/y. This value is slightly higher than the one (13.24/y) used by Ainsworth et al. [81] who based their value on an average for 5 species including *Octopus cyanea* used by Van Heukelen in Hawai`i*.* Parrish et al. [82] had a value for the combined squid and octopus group, so we used their estimate of 12/y.

The P/B ratio was calculated based on a maximum age and weight of *O. cyanea* [80]. Our value of 3.313/y was slightly higher than the one used by Ainsworth et al. [21] of 2.327/y and similar to the one used by Tsehaye and Nagelkerke [7] of 3.5/y. As we didn’t have any value for squids, we used the combined value from Parrish et al. [82] which was very similar to the one we calculated for octopus (3.5/y).

Octopus is well sought after in recreational fishery either as food or as bait [83]. From commercial landing records, 0.0085 t/km^2^ is caught annually. It was not reported in the recreational landings (only records for fish). Based on mean annual reported landings from 2006 to 2010 of both recreational and commercial fishery, the reported commercial catch is 3-5 times higher than the recreational fishery. We used a correction factor of 5 to calculate the octopus catch from recreational fishery resulting in a total fishery yield of 0.051 t/km^2^. Standing stock was estimated at 1.23 t/km^2^ resulting in an F of 0.04 per year.

S1.20 Benthic Grazers

Urchins are conspicuous invertebrates on coral reefs and changes in their abundances can have large-scale effects on reef community structure, e.g., the loss of Diadematids led to increase in macroalgae standing stock [84] and led to higher trophic level changes [85]. Diadematids (*Echinothrix* spp.) are generalists in terms of diet which makes them very resilient to changes in environmental conditions that lead to shifts in community composition. Diadematids are browsers and their preferred diet is turf algae (filamentous algae), but in absence or low abundance of that they feed on CCA, some detritus, boring algae, foraminiferans [53,86]. *Tripneustes* spp. are herbivorous and detritivorous and they graze algae [86]; the composition of their diet typically reflects the algal distribution found on the reef [87]. The short-spined *Echinometra* spp. eat boring cyanobacteria and, in doing so, erode the reef substratum which explains why calcium carbonate sediments are usually the largest fraction of the gut content. They are often found in branching corals and in burrows in the reef substratum. Drift algae can be an important part of their diet [86]. *Heterocentrotus* spp. are mostly herbivorous, grazing on filamentous or fleshy algae from bare substrate or the coral surface [86]. Juvenile echinoids are too small to browse and tend to be detritivores or grazers of encrusting algae [86].

On the Kona Coast, 0.035 urchins/m^2^ were recorded on reefs at depths of 0-21 m [88] and 0.95 ind/m^2^ at 10-m *Porites* reefs [86]. Coral-reef echinoids decrease in abundance with depth, likely related to wave stress and food availability [88]. Pacific RAMP invertebrate REA surveys conducted from 2002 to 2010 identified the following urchin species: *Echinometra* sp., *Echinostrephus* sp., *Tripneustes* sp., *Echinothix* sp., Diadema sp., and *Heterocentrotus*; other echinoids comprised less than 1% of the total abundance. At all three study sites, boring urchins (*Echinometra mathaei*, *Echinostrephus* sp.) were more prominent than the free-roaming urchin species. Based on intensive surveys for size classes in 2008, we calculated the weighted mean test size for each study site. Test diameter was converted to weight using the relationship W= 0.247·D^2.66^ [89]. On reefs around O`ahu *Tripneustus gratilla*, collectors urchin, was the most abundant (44.6% of total) of the free roaming species. Towed-diver surveys conducted in 2008 and 2010 estimated an abundance of 0.029 ind/m^2^ for boring urchins and 0.020 ind/m^2^ for free urchins. Site-specific REA surveys conducted from 2002 to 2010 showed a much higher abundance of 2.18 ind/m^2^ for boring urchins and 0.36 ind/m^2^ for free urchins. We took the mean of the two survey methods for our estimate and calculated the weighted mean biomass. On reefs around FFS, towed-diver surveys conducted from 2002 to 2010 estimated the abundance of boring urchins at 0.014 ind/m^2^ and free urchins at 0.056 ind/m^2^; REA surveys conducted in 2008 estimated 3.60 ind/m^2^ for boring urchins and 0.44 ind/m^2^ for free urchins. The most abundant species was *Echinotrix* sp. Again, we took the mean of the two methods and calculated the weighted mean biomass. Urchin data from Kona came from DAR; however, they do not survey for boring urchins so we supplemented that data with Pacific RAMP REA surveys conducted from 2002 to 2010 around Hawai`i, as those surveys estimated a similar mean abundance for the free urchins as the DAR data on the Kona Coast. *Tripneustes gratilla* made up 69% of the total free-urchin composition.

The P/B and Q/B ratios for free urchins (based on *Tripneustus* sp.*)* were taken from Wabnitz et al. [3] and for boring urchins (based on *Echinometra* sp.) Q/B was based on consumption rate reported in Appana and Vuki [90] and P/B was calculated using the empirical relationship from Brey [58] based on the weighted mean of the test of 11.23 g for Hawaiian reefs.

S1.21-1.31 Fish

For the EwE models, fish are somewhat subjectively grouped based on their functional role in the reef ecosystem, and their diet, body shape, and habitat preference to allow representation of important commercial, social, ecological, and management interest. These important specializations were determined based on ecological literature available for coral reef fish [91-95] and discussions with resource managers and coral reef fish specialists (Bill Walsh, Russell Sparks, Ed Demartini, Ivor Williams). Species assignments to the different functional groups were based on various published sources [93,96,97-99], FishBase ([www.fishbase.org](http://www.fishbase.org)), and expert opinion. Coral reefs in Hawai`i, as elsewhere in the world, exhibit phase shifts from coral to macroalgal dominance [100,101]. Therefore, we further split up the herbivore fish group based on their role in preventing this phase shift from happening and in promoting resilience of the reef. Surgeonfish and some damselfish species (denuders or grazers) and parrotfish and urchins (scrapers and excavators) play a crucial role in preventing macroalgae (i.e., all foliose algal species < 1 cm standing stock) from emerging from grazed algal turf (all foliose algal species > 1 cm) by their high feeding rate on turf [97,102] and so prevent coral overgrowth and shading by macroalgae [91]. Yet they can only maintain the reef in a cropped state if coral cover does not decrease [103,104] and they are not very effective in the reversal of a phase shift [102]. Browsers are the species that prefer to feed on macroalgal stands and could play a crucial role in the reversal of a phase shift. Excavators, and to a lesser degree scrapers, are those species that have a functional role in bioerosion, by scraping off (dead) coral and sediment and so facilitate coral and coralline algal recruitment [91]. These last two groups consist entirely of parrotfishes and the bigger they are the larger their bites so the higher their effectiveness [105,106]. However, live coral is not a significant part of the diet of parrotfish species in Hawai`i [105] unlike the *Bolbometopon muricatum* in the Indo-Pacific [98], *Chorurus gibbus* in the Red Sea [107] and *Sparisoma viride* in the Caribbean [106]

About 260 reef fish species can be seen regularly during daylight (scuba) dives (Table S2) in Hawai`i. These species do not include cryptic species and only those nocturnal species that hide in the shallow waters under overhangs or in rock crevices are easily visible to divers (CRED unpubl. data).

Fish abundance data came from random surveys stratified by 3 depth strata (shallow < 6 m, mid depth between 6 and 18 m, and deep > 18 m) conducted by the Pacific RAMP and for the Kona Coast of Hawai`i from belt-transect surveys conducted by DAR from 2005 to 2010. Mean annual site abundance values were pulled up by island and then averaged to obtain a mean abundance estimate for each study site.

From stratified random surveys conducted by DAR at 3 sites off the Kona Coast, overall biomass was comparable among the 4 depth strata which is in correspondence with the observations from the Pacific RAMP surveys and Friedlander et al. [108]. However, when looking at functional groups, it appeared that herbivores were 1.5 times more abundant in the shallow waters, piscivores were almost 3 times more abundant in the deeper waters, and planktivores were almost absent from shallow waters (26 times more abundant in deeper waters). When comparing the stratified random survey results of the shallow water biomass per functional group with 145 random timed-swim surveys, herbivores accounted for almost half the biomass estimate from the stratified random survey results. Planktivores and piscivores had comparable biomass estimates between the random swim and random stratified surveys. Data collected by Pacific RAMP showed similar patterns. By using the results of the stratified random surveys averaged over the 3 depth strata, we assume we have a good representation of fish biomass for the entire reef area of 0-30 m depth.

Diet data came from literature [92,98,99,105,109-120] and Fishbase. Diet data was compared with Ainsworth et al. [21] for a reef system in Indonesia, with Gribble [6] for a reef system on the Great Barrier Reef, Australia, and with Wabnitz et al. [3] for a reef system on the Kona Coast of Hawai`i. Ainsworth et al. [21] used a diet algorithm based on cohabitation, predator gape and prey body size [121] and we fine-tuned our diet results based on their results when averaging per functional group.

For each study area, the weighted P/B and Q/B ratios were calculated using data from literature or empirical relationships.

S1.21 Planktivores (FPL)

In total, 41 obligate and facultative planktivorous species were included in this functional group. For the Kona model, the most abundant species in this functional group in terms of biomass were the epaulette soldierfish (28.5%; *Myripristis kuntee*), a chromis species (15.7%; *Chromis agilis*), and the black triggerfish (15.1%; *Melichthys niger*). Total catch landings were estimated at 0.014 t/km^2^/y. For the O`ahu model, the most abundant species in terms of biomass were the sleek unicornfish (37.7%; *Naso hexacanthus*), the black triggerfish (28.6%; *Melichthys niger*), and a chromis species (9.9%; *Chromis vanderbilti*). Total catch landings were estimated at 0.050 t/km^2^/y. On the reef around FFS, the majority of the plantivores comprised of the black triggerfish (36.7%; *Melichthys niger*), the sleek unicornfish (19.1%; *Naso hexacanthus*), and the big scales soldierfish (16.5%: *Myripristinae berndti*).

S1.22 Coralivores (FCO)

Ten species of coralivores were included in the model of which seven belonged to the butterflyfishes, *Chaetodon* sp., a pufferfish, *Arothron meleagris*, a blenny, *Exallias brevis*, and the blue-eyed damselfish *Plectroglyphidodon johnstonianus.* In the Kona system the most abundant species in terms of biomass were the ornate and peddled butterflyfish (41.3%, *Chaetodon ornatissimus*; 31.5%, *C. multicinctus*). On the reef around O`ahu, the pufferfish was most abundant (33.3%), followed by the peddled (21.2%) and ornate (16.8%) butterfly fish and the blue-eyed damselfish (16.4%). On the FFS reefs, the species composition of coralivores was dominated in terms of biomass by the Chevron butterflyfish (71.3%, *C. trifascialis*) followed by the ornate (23.3%) and peddled (13.4%) butterflyfishes.

There was no reported recreational fishery for coralivores and reported commercial fishery was only from the aquarium trade with 0.007 t/km^2^/y for Kona and 0.003 t/km^2^/y for O`ahu.

S1.23 Invertivores (FIV)

The functional group invertivores included the highest number of species from all functional groups with 86 species and 3 additional families. For the Kona reef system, the most abundant species was a goatfish (*Mulloidichthys vanicolensis)* with 19.9%; all other species made up less than 10% of the total biomass. Around O`ahu, triggerfishes were most abundant with *Sufflamen fraenatum* accounting for 18.0% and *S. bursa* for 14.5%. The only other species with an abundance > 10% was the saddle wrasse, *Thalassoma duperrey*, with 12.4%. In FFS, the most abundant species was the introduced blue-stripe snapper or ta’ape (*Lutjanus* *kasmira*) which accounted for 41.8% of the total invertivores’ biomass followed by the big-eyed emperor (*Monotaxis grandoculis*) with 10%, the rest of the species was less than 10%.

Fishermen targeted 31 out of 86 species within this functional group (DAR unpubl. data). Total fishing mortality for this functional group was estimated at 0.08 t/km^2^/y for Kona and 0.21 t/km^2^/y for O`ahu.

S1.24 Browsers (FHB)

Nine species of browsers and one entry at genus level for chubs (*Kyphosus*) were included in this functional group. The orange spine unicornfish, *Naso lituratus*, was by far the most abundant in terms of biomass in the two reef ecosystems in the main Hawaiian Islands, accounting for 91.0% of the total browser’s biomass in the Kona system, 85% in O`ahu. In FFS, the blue-spine unicornfish (39.1%, *N. unicornis*) was slightly more abundant than the orange spine unicornfish (31.7%) followed by chubs (*Kyphosus* sp., 17.6%).

Total fishing mortality was 0.005 t/km^2^/y for Kona and 0.024 t/km^2^/y for O`ahu.

S1.25 Grazers/Detritivores (FHG)

Thirty species of grazers were included in this functional group that consisted mostly of surgeonfishes (Acanthuridae) and some tobys (Tetraodontidae), damselfishes (Pomacanthidae) and a triggerfish (*Melichthys vidua*). On the reefs along the Kona Coast, the most abundant species in terms of biomass were goldring surgeonfish (42.7%, *Ctenochaetus strigosus*) and yellow tang (34.2%, *Zebrasoma flavescens*). For the reefs around O`ahu, the most abundant species was orangeband surgeonfish (51.8%, *Acanthurus olivaceus*); other abundant species were pinktail durgeon (17.1%, *Melichthys vidua*) and brown surgeonfish (15.1%, *Acanthurus nigrofuscus*).

According to commercial catch records, surgeonfish make up a high number of total catch with a total statewide mean annual catch of 92,142 kg (2006-2010) and reported statewide recreational landings of convict tang (manini, *Acanthurus triostegus*) were also high with 13,834 kg. Total fishing mortality was 0.053 t/km^2^/y for Kona and 0.13 t/km^2^/y for O`ahu.

S1.26 Scrapers (FHS)

Scrapers consisted of two species of small-bodied parrotfishes, *Scarus dubius* and the more abundant *S. psittacus* (94.4% of total scraper biomass in Kona and 97.6% in O`ahu).

There were no reported values for commercial or recreational fishery. However, parrotfishes are found in much higher numbers in marine protected areas closed to fishing than in the open access areas, suggesting that parrotfishes are indeed fished [122]. We, therefore, included a recreational fishery landing of 0.022 t/km^2^/y in both the Kona and the O`ahu models based on the catch results of parrotfish from daytime creel surveys [83].

S1.27 Excavators (FHE)

The group of herbivorous excavators consisted of three species of parrotfishes, two of the genus *Chlorurus*: bullethead parrotfish, *C*. *sordidus,* and spectacled parrotfish, *C. perspicillatus*, and redlip parrotfish, *Scarus rubroviolaceus*. In each of the systems, a different parrotfish species was most abundant; in Kona it was the bullethead (91.9%), in O`ahu the redlip (72.6%) and in FFS the spectacled (52.0%) parrotfish.

Commercial catch data recorded a statewide mean of 49 ton/yr for Scaridae. Of the parrotfish catch, *S. rubroviolacaceus* is a preferred target species [123]. There was no reported recreational catch data. However, parrotfishes are found in much higher numbers in nearshore marine protected areas closed to fishing than in the open access areas, suggesting that parrotfishes are indeed fished [122]. We, therefore, included a recreational fishery landing that was the same as the mean statewide commercial landing for Scaridae resulting in a total fishing mortality of 0.22 t/km^2^/y in Kona and 0.25 t/km^2^/y in the O`ahu model.

S1.28 Benthic piscivores (FPB)

We included 19 species in the benthic piscivore functional group, including the introduced peacock grouper, eels, cornetfish, trumpetfish, scorpionfish, lizardfish, one wrasse and one goatfish. The peacock grouper or roi, *Cephalopholis argus*, was introduced to Hawai`i in the late 1950s to boost fisheries [124]. After a time lag of limited population growth, it was rare in the 1970s and 1980s [124] the population increased strongly and the fish became a resident predator in nearshore reefs (W. Walsh, pers. comm. 16 September 2011). However, due to the accumulation of ciguatoxin, it never became a game fish [114] and now is the most abundant benthic piscivore on some reefs (CRED unpubl. data). In Kona and O`ahu, roi was by far the most abundant benthic piscivore making up 63.5% and 59.5% respectively of the total biomass estimate. In FFS, the ringtail wrasse (46.8%, *Oxycheilinus unifasciatus*) was the most abundant in terms of biomass followed by the moray eel (27.8%, *Gymnothorax javanicus* ) and blue goatfish (21.4%, *Parupeneus cyclostomus*).

Fishing mortality was estimated at 0.002 t/km^2^/y for Kona and 0.003 t/km^2^/y for O`ahu based on commercial and recreational fishery statistics targeting five species from this functional group.

S1.29 Mid-water piscivores (FPM)

Mid-water piscivores were represented by needle- and houndfish, Heller’s barracuda, and the small tooth jobfish (*Aphareus furca*, Lutjanidae). In Kona, Heller’s baracuda (56.4%, *Sphyraena helleri*) followed by small tooth jobfish (33.0%, *Aphareus furca*) accounted for the majority of the total mid-water piscivores’ biomass. In O`ahu this was made up by small tooth jobfish (79.0%). In FFS the small tooth jobfish was the only mid-water piscivore observed during the visual surveys.

Fishing mortality was estimated at 0.022 t/km^2^/y for Kona and 0.039 t/km^2^/y for O`ahu based on commercial and recreational fishery statistics targeting three species from this functional group.

S1.30 Roving piscivores (FPR)

Roving piscivores were predominantly represented by jacks (8 species), grey snapper (*Aprion virescens*), and the great barracuda (*Sphyraena barracuda*)*.* In Kona, the most abundant species in terms of biomass was the bluefin trevally (38.3%, *Caranx melampygus*) and the greater amber jack (33.3%, *Seriola dumerili*). In O`ahu, the giant trevally (69.4%, *Caranx ignoblis*) was the most abundant as was the case in FFS (62.2%).

Mean statewide recreational landing was highest for bluefin trevally with 157,786.6 kg per year and commercial landing was highest for giant trevally with 8546 kg per year. Total fishing mortality was 0.28 t/km^2^/y for Kona and 0.44 t/km^2^/y for O`ahu; both were mostly made up by the high recreational catch records.

As a result of the high total mortality (sum of natural and fishing mortality) which is the same as the input parameter P/B, the P/Q ratio was unrealistically high (0.5) for the O’ahu model, and we, therefore, reduced P/B to 1 per year. We then had to double the biomass to mass balance the model.

S1.31 Sharks (SHR)

Five species of sharks were included in the models of which scalloped hammerhead (*Sphyrna lewini*) was most abundant in terms of biomass in Kona (92.7%), whitetip reef shark (*Triaedon obesus*) was the only species encountered in O`ahu, and gray reef shark (57.2%, *Carcharhinus amblyrhynchos*) and whitetip reef shark (32.3%) were the two most abundant species in FFS.

There were no recorded landings of reef sharks in either fishery but creel surveys did report shark landings of 0.055 t/km^2^. We assumed that this value was the same as the average annual recreational landing and used a correction factor based on the contribution of the island-based fishery to the commercial statewide fishery, i.e., 50% of the commercial landings come from O’ahu and 5.5% from Kona. This resulted in a total landing of 0.003 t/km^2^/y for Kona and 0.028 t/km^2^/y for O`ahu.

Just as for the roving piscivores, the high total mortality resulted in an unrealistic high P/Q ratio (0.8) for the O’ahu model, and we reduced P/B to 1 per year. Again, we then had to double the biomass to mass balance the model.

S1.32 Marine Reptiles

The most abundant sea turtle in Hawai`i is the green turtle, *Chelonia mydas*, with foraging green turtles along all coastlines except for Kahoolawe and the west coast of Lanai (Balazs, pers. comm., May 28, 2011). Population estimate is 42 turtles/km^2^ along 50% of the coast with an estimated total number of 61,000 green turtles [125]. The great majority of green turtles are juveniles, especially along the Kona Coast of Hawai`i (M. Weijerman, pers. obs) with a straight carapace length of around 60 cm and a mean body weight of 27.6 kg (NOAA NMFS Marine Turtle Research Program & NPS unpubl. data).

The Hawaiian green turtles use the French Frigate Shoals as their breeding grounds with 90% of all reproduction taking place there [126]. Breeding season is from May to August with 1 to 6 clutches (mean 1.8) per season [127]. Green turtles leave pelagic habitats at a carapace length of 35 cm and shift from a more omnivorous diet to a herbivorous diet, feeding primarily on macroalgae and turf although they can also, unintentionally, consume invertebrates [127].

We used the same P/B and Q/B ratios for all three models. The P/B (0.142/y) ratio is the mean from values published by Wabnitz et al. [3], Parrish et al. [82] and Polovina [4]. The Q/B ratio (6.215/y) was based on the consumption of *Thalassia* [128] and is in close agreement with Q/B ratios given by Wabnitz et al. [3] and Parrish et al. [82].

S1.33 Marine Mammals

Of the marine mammals passing through the reefs in Hawai`i, we only included the endangered Hawaiian monk seal as that species actively forages on reef fish and has, therefore, stronger ties with the modeled reefs than species like spinner dolphins that mostly use bays to rest and forage offshore or humpback whales that do not feed.

Abundance estimates from the main Hawaiian Islands were 113 individuals in 2008 [129] of which 2 were seen on the Kona Coast (T. Wurth, pers. comm.) and 12 around O`ahu. Mean weight for the different age categories at FFS was: pup 0-2 yr, 60 kg; juvenile 3-4 yr 125 kg, subadult 5+yr, 170 kg which gave us a weighted mean of 84.6 kg for juveniles [130]. Average weight for adults is 187.5 (range between 180 and 270 kg with females being slightly larger [131]. Immature (pups, juveniles and subadults) animals made up 56% of the population in 2001. Using these estimates we calculated the biomass for Kona and O`ahu. For FFS, we used the biomass reported from FFS [82].

Diet data for monk seals in the NWHI was based on the prey type and dominance identified in a quantitative fatty acid signature analysis [132]). A reference library of more than 100 fish taxa (*n* = 2,190) collected was used to evaluate the signatures of monk seal blubber samples and indicated that the seals’ diet was roughly made up of 12% reef fish, 67% mesophotic prey, and 21% subphotic prey [132]. In the MHI diet, composition was slightly different [132]. To account for the movement of monk seals outside of the modeled area boundaries to forage, we represented part of their feeding ecology as occurring outside the system by setting 57% of their diet as ‘import’ in the Ecopath diet composition matrix.

P/B (0.1/y) and Q/B (8/y) ratios were taken from an Ecopath model focusing on Hawaiian monk seals in the Northwestern Hawaiian Islands [82]. Pups in the MHI are in better condition [130] and, therefore, have a higher survival rate so we assumed a P/B ratio of 0.2/y for the O`ahu and Kona model.

Ecopath input and output parameters

Tables S3, S4 and S5 give the input parameters as described above and some of the output parameters calculated by Ecopath (all in bold) for each study area.

Ecopath diet matrices

Tables S6, S7 and S8 show the Ecopath diet composition matrices for the functional groups (details in Supplementary text S1) in the balanced models for the three reef systems.

S4. **Fishery**

The annual mean catch of reef fish from commercial fishery statistics for the entire state was 213,066 kg for the main Hawaiian Islands between 2006 and 2010 (www.pifsc.noaa.gov/wpacfin/hi/dar/Pages/hi_data_3.php. Accessed 2011 Jan. During the same time period, mean annual reported recreational yield of reef species was 371,824 kg, which is 1.8 times more than the commercial catch underlining its importance in the total fishery. The aquarium fishery also takes many fishes (394,865 individuals annually). As they are generally small juveniles, we estimated the total annual catch to be only 3554 kg/yr for FY 2006-2010 assuming an average size of 7.5 cm and using the length-weight relationship of the yellow tang (*Zebrasoma flavescens*) as that species made up the 82% of the annual catch in numbers.

Creel surveys were conducted in Hanalei, Kauai, Kane`ohe, O`ahu [83,133] and Puako, Hawai`i (J. Giddens, pers. comm.). For Hanalei Bay, total catch (excluding small pelagics) was 46,822 kg comprising 95 taxa; in contrast, the reported commercial catch, including offshore as well as inshore fishing, on the north shore of Kaua`i encompassing Hanalei Bay was only 5490 kg comprising 28 taxa. Another example of the discrepancy between the reported catch and creel surveys is the harvest of octopi; average annual commercial landings during 1980-1990 were 5818 kg/y, whereas results from the creel survey (1991-1992) showed that in Kane`ohe alone annual catch was 13,618 kg, more than twice the annual statewide reported catch.

The overall yield for Kane`ohe was 0.92–1.4 t/km^2^/y and for Hanalei 0.8 t/km^2^/y, whereas the overall yield from the reported recreational landings was only 0.3 t/km^2^/y. In the state of Hawai`i there is no requirement for recreational or subsistence fishers to report their catch [100]. However, based on the above results, it is clear that total fishery harvest is likely around 3-10 times higher for most targeted taxa than the provided recreational catch [134]. We used observed discrepancies between the results of creel surveys and reported landings of the recreational fishery to generate taxon-specific calibration factors to estimate ‘corrected’ catch values for recreational landings. The yield of goatfish, surgeonfish, and chubs calculated from recreational fishery was about 2, 3 and 10 times lower compared to the yield of those species from the creel surveys, so we multiplied the recreational landings data of those three taxa with their respective correction factor. Shark and parrotfish landings were not mentioned in the recreational fishery data. However, creel data showed that these species were targeted by recreational fishermen. Based on the average landings from the creel surveys, we included a recreational catch of 0.055 t/km^2^ for reef sharks, 0.2 t/km^2^ for large-bodies parrotfish species (excavators) and 0.02 t/km^2^ for small-bodies parrotfish species (scrapers).

S5. REFERENCES

1. Christensen V, Pauly D (1993) Trophic models of aquatic ecosystems. ICLARM Conference Proceedings 26.

2. Polovina JJ, Chai F, Howell EA, Kobayashi DR, Shi L, et al. (2008) Ecosystem dynamics at a productivity gradient: A study of the lower trophic dynamics around the northern atolls in the Hawaiian Archipelago. Progress In Oceanography 77:217-224.

3. Wabnitz CCC, Balazs G, Beavers S, Bjorndal KA, Bolten AB, et al. (2010) Ecosystem structure and processes at Kaloko Honokohau, focusing on the role of herbivores, including the green sea turtle Chelonia mydas, in reef resilience. Marine Ecology Progress Series 420:27-44.

4. Polovina JJ (1984) Model of a coral reef ecosystem. Coral Reefs 3:1-11.

5. Grigg RW, Polovina JJ, Atkinson MJ (1984) Model of a coral reef ecosystem. Coral Reefs 3:23-27.

6. Gribble N (2003) GBR-prawn: modelling ecosystem impacts of changes in fisheries management of the commercial prawn (shrimp) trawl fishery in the far northern Great Barrier Reef. Fisheries Research 65:493-506.

7. Tsehaye I, Nagelkerke LAJ (2008) Exploring optimal fishing scenarios for the multispecies artisanal fisheries of Eritrea using a trophic model. Ecological Modelling 212:319-333.

8. Hatcher B (1997) Organic production and decomposition. In: Birkeland C, editor. Life and death of coral reefs. New York. Chapman and Hall. pp. 140-174.

9. Opitz S (1996) Trophic interactions on a Caribbean coral reef. Tech. Rep. 43. Manila, Philippines. ICLARM. 341 p.

10. Bienfang P, Szyper J, Okamoto M, Noda E (1984) Temporal and spatial variability of phytoplankton in a subtropical ecosystem. Limnology and Oceanography29:527-539.

11. Roman MR, Adolf HA, Landry MR, Madin LP, Steinberg DK, et al. (2001) Estimates of oceanic mesozooplankton production: a comparison using the Bermuda and Hawai`i time-series data. Deep Sea Research Part II: Topical Studies in Oceanography 49:175-192.

12. Karl DM, Bidigare RR, Letelier RM (2001) Long-term changes in plankton community structure and productivity in the North Pacific Subtropical Gyre: The domain shift hypothesis. Deep-Sea Research Part Ii-Topical Studies in Oceanography 48:1449-1470.

13. Heidelberg KB, O'Neil KL, Bythell JC, Sebens KP (2010) Vertical distribution and diel patterns of zooplankton abundance and biomass at Conch Reef, Florida Keys (USA). Journal of Plankton Research 32:75-91.

14. Genin A, Monismith SG, Reidenbach MA, Yahel G, Koseff JR (2009) Intense benthic grazing of phytoplankton in a coral reef. Limnology and Oceanography 54:938-951.

15. Chardy P, Clavier J. An attempt to estimate the carbon budget for the southwest lagoon of New Caledonia; 1988; Australia. pp. 541-546.

16. Fauchald K, Jumars PA (1979) The diet of worms: a study of polychaete feeding guilds. Oceanography and Marine Biology: An Annual Review 17:193-284.

17. Heidelberg K, Sebens K, Purcell J (2004) Composition and sources of near reef zooplankton on a Jamaican forereef along with implications for coral feeding. Coral Reefs 23:263-276.

18. Alldredge A, King J (2009) Near-surface enrichment of zooplankton over a shallow back reef: implications for coral reef food webs. Coral Reefs 28:895-908.

19. Calbet A, Landry MR, Scheinberg RD (2000) Copepod grazing in a subtropical bay: species-specific responses to a midsummer increase in nanoplankton standing stock. Marine Ecology Progress Series 193:75-84.

20. Le Borgne R (1982) Zooplankton production in the eastern tropical Atlantic Ocean: Net growth efficiency and P:B in terms of carbon, nitrogen, and phosphorus. Limnology and Oceanography 27:681-698.

21. Ainsworth C, Varkey D, Pitcher T (2007) Ecosystem simulation models for the Bird’s Head Seascape, Papua, fitted to field data. In: Pitcher TJ, C.H A, Bailey M, editors. Ecological and economic analyses of marine ecosystems in the Bird’s Head Seascape, Papua, Indonesia. I: Fisheries Centre Research Reports. pp. 6–172.

22. Smith JE, Smith CM, Hunter CL (2001) An experimental analysis of the effects of herbivory and nutrient enrichment on benthic community dynamics on a Hawaiian reef. Coral Reefs 19:332-342.

23. Klumpp D, McKinnon A (1992) Community structure, biomass and productivity of epilithic algal communities on the Great Barrier Reef: Dynamics at different spatial scales. Marine Ecology Progress Series 86:77-89.

24. Miller RJ, Reed DC, Brzezinski MA (2009) Community structure and productivity of subtidal turf and foliose algal assemblages. Marine Ecology Progress Series 388:1-11.

25. Carpenter RC, Hackney JM, Adey WH (1991) Measurements of primary productivity and nitrogenase activity of coral reef algae in a chamber incorporating oscillatory flow. Limnology and Oceanography36:40-49.

26. Littler MM, Murray SN (1974) The primary productivity of marine macrophytes from a rocky intertidal community. Marine Biology 27:131-135.

27. Hatcher BG (1990) Coral reef primary productivity. A hierarchy of pattern and process. Trends in Ecology & Evolution 5:149-155.

28. Marsh Jr JA (1970) Primary productivity of reef-building calcareous red algae. Ecology:255-263.

29. Anthony KRN, Kline DI, Diaz-Pulido G, Dove S, Hoegh-Guldberg O (2008) Ocean acidification causes bleaching and productivity loss in coral reef builders. Proceedings of the National Academy of Sciences 105:17442-17446.

30. Odum HT, Odum EP (1955) Trophic structure and productivity of a windward coral reef community on Eniwetok Atoll. Ecological Monographs25:291-320.

31. Hatcher BG (1988) Coral reef primary productivity: a beggar's banquet. Trends in Ecology & Evolution 3:106-111.

32. Payri CE (1988) Halimeda contribution to organic and inorganic production in a Tahitian reef system. Coral Reefs 6:251-262.

33. Birkeland C (1987) Nutrient availability as a major determinant of differences among coastal hard-substratum communities in different regions of the tropics. UNESCO Reports in Marine Science.

34. Smith SV, Kimmerer WJ, Laws EA, Brock RE, Walsh TW (1981) Kaneohe Bay Sewage Diversion Experiment: Perspectives on Ecosystem Responses to Nutritional Perturbation l. Pacific Science 35:279-395.

35. Wilkinson CR, Cheshire AC (1990) Comparisons of sponge populations across the Barrier Reefs of Australia and Belize. Evidence for higher productivity in the Caribbean. Marine Ecology Progress Series 67:285-294.

36. Yahel G, Sharp JH, Marie D, Häse C, Genin A (2003) In situ feeding and element removal in the symbiont-bearing sponge *Theonella swinhoei*: Bulk DOC is the major source for carbon. Limnology and Oceanography 48:141-149.

37. Yahel G, Zalogin T, Yahel R, Genin A (2006) Phytoplankton grazing by epi-and infauna inhabiting exposed rocks in coral reefs. Coral Reefs 25:153-163.

38. Fabricius KE, Dommisse M (2000) Depletion of suspended particulate matter over coastal reef communities dominated by zooxanthellate soft corals. Marine Ecology Progress Series 196:157-167.

39. Goffredo S, Lasker H (2008) An adaptive management approach to an octocoral fishery based on the Beverton-Holt model. Coral Reefs 27:751-761.

40. Veron JEN, Stafford-Smith MG (2002) Coral ID. Australian Institute of Marine Science: CD-ROM.

41. Forsman ZH, Concepcion GT, Haverkort RD, Shaw RW, Maragos JE, et al. (2010) Ecomorph or endangered coral? DNA and microstructure reveal Hawaiian species complexes: *Montipora dilitata/flabellata/turgescens* & *M. patula/verrilli*. PLoS One 5:e15021.

42. Palardy JE, Rodrigues LJ, Grottoli AG (2008) The importance of zooplankton to the daily metabolic carbon requirements of healthy and bleached corals at two depths. Journal of Experimental Marine Biology and Ecology 367:180-188.

43. Palardy JE, Grottoli AG, Matthews KA (2006) Effect of naturally changing zooplankton concentrations on feeding rates of two coral species in the Eastern Pacific. Journal of Experimental Marine Biology and Ecology 331:99-107.

44. Kroeker KJ, Micheli F, Gambi MC, Martz TR (2011) Divergent ecosystem responses within a benthic marine community to ocean acidification. Proceedings of the National Academy of Sciences 108:14515-14520.

45. Choy SC (1986) Natural diet and feeding habits of the crabs *Liocarcinus puber* and *L. holsatus* (Decapoda, Brachyura, Portunidae). Marine Ecology Progress Series 31:87-99.

46. Jernakoff P, Phillips B, Fitzpatrick J (1993) The diet of post-puerulus western rock lobster, *Panulirus* *cygnus* George, at Seven Mile Beach, Western Australia. Marine and Freshwater Research 44:649-655.

47. Malaquias MAE, Condinho S, Cervera JL, Sprung M (2004) Diet and feeding biology of *Haminoea* *orbygniana* (Mollusca: Gastropoda: Cephalaspidea). Journal of the Marine Biological Association of the UK 84:767-772.

48. Mayfield S, Atkinson LJ, Branch GM, Cockcroft AC (2000) Diet of the West Coast rock lobster *Jasus* *lalandii*: influence of lobster size, sex, capture depth, latitude and moult stage. South African Journal of Marine Science 22:57-69.

49. Stehlik LL (1993) Diets of the brachyuran crabs *Cancer irroratus*, *C. borealis*, and *Ovalipes ocellatus* in the New York Bight. Journal of Crustacean Biology 13:723-735.

50. Yonow N (1992) Observations on the diet of *Philinopsis cyanea* (Martens) (Cephalaspidea: Aglajidae). Journal of Conchology 34:199.

51. Hoover JP (1999) Hawai`i's sea creatures; A guide to Hawai`is marine invertebrates. Honolulu, Hawai`i. Mutual Publishing.

52. McLaughlin PA, Bailey-Brock JH (1975) A new Hawaiian hermit crab of the genus *Trizopagurus* (Crustacea, Decapoda, Diogenidae), with notes on its behavior. Pacific Science 29:259-266.

53. Uthicke S, Schaffelke B, Byrne M (2009) A boom-bust phylum? Ecological and evolutionary consequences of density variations in echinoderms. Ecological Monographs 79:3-24.

54. Monteforte M (1987) The decapod Reptantia and stomatopod crustaceans of a typical high island coral reef complex in French Polynesia (Tiahura, Moorea Island): zonation, community composition and trophic structure. Atoll Research Bulletin 309:1-37.

55. Enochs IC (2010) Motile Cryptofauna of an Eastern Pacific Coral Reef: Biodiversity and Trophic Contribution. Miami, FL. University of Miami. 253 p.

56. Enochs IC, Toth LT, Brandtneris VW, Afflerbach JC, Manzello DP (2011) Environmental determinants of motile cryptofauna on an eastern Pacific coral reef. Marine Ecology Progress Series 438:105-118.

57. Cartes J, Brey T, Sorbe J, Maynou F (2002) Comparing production-biomass ratios of benthos and suprabenthos in macrofaunal marine crustaceans. Canadian Journal of Fisheries and Aquatic Sciences 59:1616-1625.

58. Brey T (2001) Population dynamics in benthic invertebrates. A virtual handbook, http://www.awi-bremerhaven.de/Benthic/Ecosystem/FoodWed/handbook/main.html . Alfred Wegener Institute for Polar and Marine Research, Germany. Available: <http://epic.awi.de/4674/>. Accessed 2011 Jan.

59. Cammen LM (1979) Ingestion rate: An empirical model for aquatic deposit feeders and detritivores. Oecologia 44:303-310.

60. Safarik M, Redden AM, Schreider MJ (2006) Density-dependent growth of the polychaete *Diopatra* *aciculata*. Scientia Marina (Barcelona).

61. Morton B (1990) The physiology and feeding behaviour of two marine scavenging gastropods in Hong Kong: the subtidal *Babylonia lutosa* (Lamarck) and the intertidal *Nassarius festivus* (Powys). Journal of Molluscan Studies 56:275-288.

62. Edwards DC, Huebner JD (1977) Feeding and growth rates of *Polinices duplicatus* preying on *Mya* *arenaria* at Barnstable Harbor, Massachusetts. Ecology 58:1218-1236.

63. McCarthy DA, Young CM, Emson RH (2003) Influence of wave-induced disturbance on seasonal spawning patterns in the sabellariid polychaete *Phragmatopoma lapidosa*. Marine Ecology Progress Series 256:123-133.

64. Mladenov P, Emson R (1984) Divide and broadcast: sexual reproduction in the West Indian brittle star *Ophiocomella ophiactoides* and its relationship to fissiparity. Marine Biology 81:273-282.

65. Glynn PW (1973) Aspects of the ecology of coral reefs in the western Atlantic region. In: Jones OA, Edndean R, editors. Biology and geology of coral reefs. New York. Academic Press. pp. 271-324.

66. Capo TR, Fieber LA, Stommes DL, Walsh PJ (2002) The effect of stocking density on growth rate and maturation time in laboratory-reared California sea hares. Journal of the American Association for Laboratory Animal Science 41:18-23.

67. Broom M (1982) Analysis of the Growth of Anadara granosa(Bivalvia: Arcidae) in Natural, Artificially Seeded and Experimental Populations. Marine Ecology Progress Series 9:69-79.

68. Werding B (1990) *Alpheus schmitti* Chace, 1972, a coral rock boring snapping-shrimp of the tropical western Atlantic (Decapoda, Caridea). Crustaceana 58:88-96.

69. Harikrishnan M, Unnikrishnan U, Maju MS, Greeshma ARR, Kurup BM (2010) Size at sexual maturity, egg number and reproductive output of the snapping shrimp *Alpheus euphrosyne* *euphrosyne* De Man, 1897. Invertebrate Reproduction & Development 54:195-202.

70. Pauly D (1980) On the interrelationships between natural mortality, growth parameters, and mean environmental temperature in 175 fish stocks. Journal du Conseil 39:175-192.

71. O'Malley JM, MacDonald CD (2009) Preliminary growth estimates of Northwestern Hawaiian Islands spiny lobster (*Panulirus marginatus*): indications of spatiotemporal variability. Honolulu, HI 96822-2396. US Department of Commerce, National Oceanic and Atmospheric Administration, National Marine Fisheries Service, Pacific Islands Fisheries Science Center. 11 p.

72. Ebert TA (1978) Growth and size of the tropical sea cucumber *Holothuria* (*Halodeima*) *atra* Jager at Enewetak Atoll, Marshall Islands. Pacific Science 32:183-191.

73. Christensen V, Walters CJ, Pauly D, Forrest R (2008) Ecopath with Ecosim version 6: user guide. November 2008. Fisheries Centre, University of British Columbia, Vancouver, Canada. pp 235.

74. Birkeland C, Lucas JS (1990) *Acanthaster planci*: major management problem of coral reefs. Boca Raton, FL. CRC Press. pp 257.

75. Yamaguchi M (1976) Estimating the length of the exponential growth phase: Growth increment observations on the coral-reef asteroid *Culcita novaeguineae*. Marine Biology 39:57-59.

76. Zann L, Brodie J, Vuki V (1990) History and dynamics of the crown-of-thorns starfish *Acanthaster* *planci* (L.) in the Suva area, Fiji. Coral Reefs 9:135-144.

77. Glynn PW, Krupp DA (1986) Feeding biology of a Hawaiian sea star corallivore, *Culcita* *novaeguineae* Muller & Troschel. Journal of Experimental Marine Biology and Ecology 96:75-96.

78. Glynn PW, Krupp DA (1986) Feeding biology of a Hawaiian sea star corallivore, *Culcita* *novaeguineae* Muller & Troschel. Journal of Experimental Marine Biology and Ecology 96:75-96.

79. Bell J (2008) Feeding preferences of the Cushion Star *Culcita novaeguineae* in the presence of the Crown of Thorns Starfish *Acanthaster planci*. Student Research Papers, UCB Moorea Class: Biology and Geomorphology of Tropical Islands, Berkeley Natural History Museum, UC Berkeley.

80. Van Heukelem WF (1976) Growth, bioenergetics and life-span of *Octopus cyanea* and *Octopus maya*. University of Hawai`i at Manoa.

81. Ainsworth C, Varkey D, Pitcher TJ (2006) Preliminary Ecosystem Simulation Models for the Bird’s Head Seascape, Papua. Mid-term narrative technical report, Birds Head Seascape Ecosystem-based Management Project. University of British Colombia Fisheries Centre. 274 p.

82. Parrish FA, Howell EA, Antonelis GA, Iverson SJ, Littnan CL, et al. (2011) Estimating the carrying capacity of French Frigate Shoals for the endangered Hawaiian monk seal using Ecopath with Ecosim. Marine Mammal Science 28:522-541.

83. Everson A, Friedlander AM (2004) Catch, effort, and yields for coral reef fisheries in Kane‘ohe Bay, O‘ahu and Hanalei Bay, Kaua‘i: Comparisons between a large urban and a small rural embayment. Status of Hawai`i’s Coastal Fisheries in the New Millennium. 110-131 p.

84. Lessios H, Robertson D, Cubit J (1984) Spread of Diadema mass mortality through the Caribbean. Science 226: 335-337.

85. Hay ME, Taylor PR (1985) Competition between herbivourous fishes and urchins on Caribbean reefs. Oecologia 65: 591-598.

86. Birkeland C (1989) The influence of echinoderms on coral-reef communities. Echinoderm studies 3.

87. Stimson J, Cunha T, Philippoff J (2007) Food preferences and related behavior of the browsing sea urchin *Tripneustes gratilla* (Linnaeus) and its potential for use as a biological control agent. Marine Biology 151:1761-1772.

88. Ebert TA (1971) A preliminary quantitative survey of the echinoid fauna of Kealakekua and Honaunau Bays, Hawai’i. Pacific Science 25:112-131.

89. Russo A (1977) Water flow and the distribution and abundance of echinoids (Genus *Echinometra*) on an Hawaiian reef. Marine and Freshwater Research 28:693-702.

90. Appana SD, Vuki VC (2006) Foraging behavior, substrate preference and influence of Echinometra sp. A on the carbonate budget of Nukubuco Reef, Fiji Islands. Micronesica-Agana 38:191.

91. Bellwood DR, Hughes TP, Folke C, Nystrom M (2004) Confronting the coral reef crisis. Nature 429:827-833.

92. Cvitanovic C, Fox R, Bellwood D (2007) Herbivory by fishes on the Great Barrier Reef: A review of knowledge and understanding. Unpublished report to the Marine and Tropical Sciences Research Facility Reef and Rainforest Research Centre Limited, Cairns, Australia. pp. 33.

93. Green AL, Bellwood DR (2009) Monitoring functional groups of herbivorous reef fishes as indicators of coral reef resilience: a practical guide for coral reef managers in the Asia Pacific Region. IUCN, Gland, Switserland. pp. 70.

94. Friedlander A, Brown E, Monaco M (2007) Defining reef fish habitat utilization patterns in Hawai`i: comparisons between marine protected areas and areas open to fishing. Marine Ecology Progress Series 351:221-233.

95. Jennings S, Kaiser MJ, Reynolds JD (2001) Marine fisheries ecology. Wiley-Blackwell. 393 p.

96. Friedlander AM, DeMartini EE (2002) Contrasts in density, size, and biomass of reef fishes between the northwestern and the main Hawaiian islands: the effects of fishing down apex predators. Marine Ecology Progress Series 230:253-264.

97. Steneck R. Herbivory on coral reefs: a synthesis; 1988. Australia. pp. 37-49.

98. Bellwood DR, Choat JH (1990) A functional analysis of grazing in parrotfishes (family Scaridae): the ecological implications. Environmental Biology of Fishes 28:189-214.

99. Choat JH, Robertson DR (2002) Age-based studies on coral reef fishes. Coral Reef Fishes: Dynamics and Diversity in a Complex Ecosystem. San Diego, Academic Press. pp. 57-80.

100. Friedlander A, Aeby G, Brainard R, Brown E, Chaston K, et al. (2008) The state of coral reef ecosystems of the main Hawaiian Islands. In: Waddell JE, Clarke AM, editors. The state of coral reef ecosystems of the United States and Pacific freely associated states: 2008. Silver Spring, MD. NOAA Technical Memorandum NOS NCCOS 73. NOAA/NCCOS Center for Coastal Monitoring and Assessment's Biogeography Team. pp. 222-269.

101. Mumby P, Hedley J, Zychaluk K, Harborne A, Blackwell P (2006) Revisiting the catastrophic die-off of the urchin *Diadema antillarum* on Caribbean coral reefs: Fresh insights on resilience from a simulation model. Ecological Modelling 196:131-148.

102. Bellwood DR, Hughes TP, Hoey AS (2006) Sleeping functional group drives coral-reef recovery. Current Biology 16:2434-2439.

103. Mumby P (2006) The impact of exploiting grazers (Scaridae) on the dynamics of Caribbean coral reefs. Ecological Applications 16:747-769.

104. Williams ID, Polunin NVC, Hendrick VJ (2001) Limits to grazing by herbivorous fishes and the impact of low coral cover on macroalgal abundance on a coral reef in Belize. Marine Ecology Progress Series 222:187-196.

105. Ong L, Holland KN (2010) Bioerosion of coral reefs by two Hawaiian parrotfishes: species, size differences and fishery implications. Marine Biology 157:1313-1323.

106. Bruggemann J, van Kessel A, van Rooij J, Breeman A (1996) Bioerosion and sediment ingestion by the Caribbean parrotfish Scarusvetula and Sparisomaviride: implications of fish size, feeding mode and habitat use. Marine Ecology Progress Series 134:59-71.

107. Alwany MA, Thaler E, Stachowitsch M (2009) Parrotfish bioerosion on Egyptian Red Sea reefs. Journal of Experimental Marine Biology and Ecology 371:170-176.

108. Friedlander AM, Brown EK, Jokiel PL, Smith WR, Rodgers KS (2003) Effects of habitat, wave exposure, and marine protected area status on coral reef fish assemblages in the Hawaiian archipelago. Coral Reefs 22:291-305.

109. Hobson ES (1974) Feeding relationships of teleostean fishes on coral reefs in Kona, Hawai`i. Fish Bull 72:915-1031.

110. Bruggemann JH, Begeman J, Bosma EM, Verburg P, Breeman AM (1994) Foraging by the stoplight parrotfish *Sparisoma viride*. 11. Intake and assimilation of food, protein, and energy. Marine Ecology Progress Series 106:57-71.

111. Sudekum A, Parrish J, Radtke R, Ralston S (1991) Life history and ecology of large jacks in undisturbed, shallow, oceanic communities. Fishery Bulletin 89:493-513.

112. Choat J (1991) The biology of herbivorous fishes on coral reefs. In: Sale PF, editor. The ecology of fishes on coral reefs. San Diego, Academic Press. pp. 120-155.

113. Choat JH, Robbins WD, Clements KD (2004) The trophic status of herbivorous fishes on coral reefs. Marine Biology 145:445-454.

114. Dierking J (2007) Effects of the introduced predatory fish *Cephalopholis argus* on native reef fish populations in Hawai`i (PhD Dissertation). Honolulu, HI. University of Hawai`i at Manoa. 125 p.

115. Meyer CG, Holland KN, Wetherbee BM, Lowe CG (2001) Diet, resource partitioning and gear vulnerability of Hawaiian jacks captured in fishing tournaments. Fisheries Research 53:105-113.

116. Guiasu RC, Winterbottom R (1998) Yellow juvenile color pattern, diet switching and the phylogeny of the surgeonfish genus *Zebrasoma* (Percomorpha, Acanthuridae). Bulletin of Marine Science 63:277-294.

117. Crossman DJ, Choat JH, Clements KD (2005) Nutritional ecology of nominally herbivorous fishes on coral reefs. Marine Ecology Progress Series 296:129-142.

118. Chen LS (2002) Post-settlement diet shift of *Chlorurus sordidus* and *Scarus schlegeli* (Pisces: Scardiae). Zoological Studies Taipei 41:47-58.

119. Paddack MJ, Cowen RK, Sponaugle S (2006) Grazing pressure of herbivorous coral reef fishes on low coral-cover reefs. Coral Reefs 25:461-472.

120. Young RF, Winn HE, Montgomery W (2003) Activity patterns, diet, and shelter site use for two species of moray eels, *Gymnothorax moringa* and *Gymnothorax vicinus*, in Belize. Copeia 2003:44-55.

121. Karpouzi V, Stergiou K (2003) The relationships between mouth size and shape and body length for 18 species of marine fishes and their trophic implications. Journal of Fish Biology 62:1353-1365.

122. Howard KG, Schumacher BD, Parrish JD (2009) Community structure and habitat associations of parrotfishes on O’ahu, Hawai`i. Environmental Biology of Fishes 85:175-186.

123. Hawai`i Cooperative Fishery Research Unit (2008) Biology of parrotfish in Hawai`i. Prepared for the Western Pacific Regional Fishery Management Council. Honolulu, Hawai`i. pp. 42.

124. Randall JE (1987) Introductions of marine fishes to the Hawaiian Islands. Bulletin of Marine Science 41:490-502.

125. Chaloupka M, Balazs G (2007) Using Bayesian state-space modelling to assess the recovery and harvest potential of the Hawaiian green sea turtle stock. Ecological Modelling 205:93-109.

126. Balazs GH, Chaloupka M (2004) Thirty-year recovery trend in the once depleted Hawaiian green sea turtle stock. Biological Conservation 117:491-498.

127. Balazs GH, Forsyth RG, Kam AKH (1987) Preliminary assessment of habitat utilization by Hawaiian green turtles in their resident foraging pastures. NOAA-TM-NWFS-SWFC-71. Honolulu, HI. National Oceanic and Atmospheric Administration, National Marine Fisheries Service, Southwest Fisheries Center. pp. 107.

128. Bjorndal KA (1997) Foraging ecology and nutrition of sea turtles. The biology of sea turtles 2. 199-231 p.

129. Baker JD, Harting AL, Wurth TA, Johanos TC (2011) Dramatic shifts in Hawaiian monk seal distribution predicted from divergent regional trends. Marine Mammal Science 27:78-93.

130. Baker JD, Johanos TC (2004) Abundance of the Hawaiian monk seal in the main Hawaiian Islands. Biological Conservation 116:103-110.

131. NOAA (2011) Learn about Monk Seals. Available: <http://www.fpir.noaa.gov/PRD/prd_hms_learn_about.html>. Accessed 2010 Dec.

132. Iverson S, Piche J, Blanchard W (2011) Hawaiian Monk Seals and Their Prey: Assessing Characteristics of Prey Species Fatty Acid Signatures and Consequences for Estimating Monk Seal Diets Using Quantitative Fatty Acid Signature Analysis. NOAA-TM-NMFS-PIFSC-23. Honolulu, HI. U.S. Dep. Commer., NOAA Tech. Memo. pp. 114 + Appendices.

133. Friedlander AM, Parrish JD (1997) Fisheries harvest and standing stock in a Hawaiian Bay. Fisheries Research 32:33-50.

134. Williams I, Walsh W, Schroeder R, Friedlander A, Richards B, et al. (2008) Assessing the importance of fishing impacts on Hawaiian coral reef fish assemblages along regional-scale human population gradients. Environmental Conservation 35:261-272.
